# Recoloring for Renewal: Preparation and Performance of Colored Slag-Based 3D Printing Materials

**DOI:** 10.3390/ma19163434

**Published:** 2026-08-13

**Authors:** Dongsheng Li, Silu Bao, Jiya Tian

**Affiliations:** School of Information Engineering, Xinjiang Institute of Technology, Aksu 843100, China; 2025146@xjit.edu.cn (D.L.); 2025110@xjit.edu.cn (S.B.)

**Keywords:** colored slag-based 3D printing materials, blast furnace slag, 3D printing, solid waste recycling

## Abstract

The current reuse of blast furnace slag is limited, and the products made from it have low added value and minimal pricing potential. The primary objective of this research is to develop new eco-friendly 3D printing materials using blast furnace slag as the main raw material, simultaneously achieving combined optimization of color appearance and material performance, to increase the reutilization value of slag and address environmental problems caused by slag. Existing studies on slag-based 3D printing materials mainly focus on improving material performance, often neglecting the combined optimization of color and material performance. This study proposes a solution to create colored slag-based 3D printing materials, aiming to break the conventional view of slag waste as simply “black or gray.” This study optimized the particle size distribution of slag-based 3D printing materials using the Andreasen model. The CIELAB color difference formula was applied to reveal how color difference values varied under different mix ratios. Digital image analysis was conducted to evaluate the color characteristics of the specimens and the uniformity of the pigmentation. After 28 days of natural air curing, the color difference ΔE at various measurement points on each colored specimen remained below 3.0, indicating that iron oxide pigments exhibit satisfactory color stability within the slag matrix. To ensure high-quality 3D printing, this study examined the effect of water temperature on the curing time of colored slag-based 3D printing materials. Range analysis results showed that water temperature exerted the most significant influence on setting time (range = 255 s), substantially greater than that of pigment dosage (range = 15 s) and pigment type (range = 5 s). The Herschel–Bulkley constitutive model was used to calculate the flow index of the material. Printing tests confirmed that colored slag 3D printing materials are suitable for extrusion-based 3D printing. The 28-day compressive test results showed that the average fracture load of the three pigmented specimen groups ranged from 23.30 to 24.58 N. Cost analysis further indicated that the comprehensive material cost is approximately 467 RMB/ton, which is lower than that of commercially available colored cement, demonstrating favorable economic competitiveness. The development of colored materials for 3D printing based on blast furnace slag can expand their applications and market potential. It also improves material performance and market acceptance, and its cost advantage over commercial colored cement further enhances its viability for practical applications, promoting high-value recycling and reuse of slag waste.

## 1. Introduction

### 1.1. Research Background

The global iron and steel industry has developed rapidly. It has generated substantial economic benefits, but it has also produced large quantities of blast furnace slag as solid waste. During ironmaking, iron ore, coke, and limestone are charged into a blast furnace in fixed proportions. The amount of slag depends on the raw materials and process conditions used in each plant. In most cases, the production of 1 ton of pig iron generates about 300–450 kg of slag. Only a few plants produce less than 300 kg per ton [1]. Blast furnace slag is currently used as a filling material in engineering works, such as embankments [2,3,4]. However, both academia and industry agree that its traditional applications, such as use as a cement admixture and subgrade fill, are mature but have low added value. These applications do not fully utilize the potential of slag and are regarded as downcycling. They also face market saturation and limited economic returns [5,6]. Therefore, current research has shifted toward the high-value utilization of blast furnace slag.

#### 1.1.1. Research on Slag-Based 3D Printing Materials

Additive manufacturing (AM), also known as 3D printing, produces objects by depositing materials layer by layer [7]. Rapid advances in this technology in recent years have enabled many eco-friendly materials to achieve higher value-added applications. Blast furnace slag exhibits hydraulicity [8,9,10] and low-carbon, eco-friendly characteristics [11,12], making it a promising material for sustainable 3D printing. Granulated blast furnace slag contains angular particles that interlock with each other. This interlocking effect improves buildability and early-age strength. As a result, the material is well-suited for 3D printing [13]. Current research on blast furnace slag in 3D printing mainly focuses on using it as a functional filler to improve material performance. For example, Panda et al. reported that adding blast furnace slag enhances the early strength of 3D-printed concrete composites [13]. Sando et al. found that adding slag powder to fly ash-based printing materials provides support and prevents collapse during printing [14]. Tseng et al. showed that concrete samples with a high content of granulated blast furnace slag exhibit better performance [15]. Juhari et al. demonstrated that optimizing slag content improves flowability, extrudability, buildability, and compressive strength of 3D-printed concrete mixtures [16]. In addition to its use as an additive, Li et al. investigated using blast furnace slag as the main material. They combined it with desulfurized gypsum to develop a new eco-friendly 3D printing material [17]. However, most studies on slag-based 3D printing materials (SB-3DPM) remain at the stage of preparing basic materials. To expand the use of slag-based 3D printing materials from structural applications into architecture and decoration, further research is required, particularly on practical properties like color expression and texture.

As an industrial solid waste, blast furnace slag often creates a stereotypical impression of “black or gray”. It is therefore important to overcome the inherent color limitations of SB-3DPM. Enhancing their visual expression and enabling personalized and artistic fabrication of printed models has clear practical value.

#### 1.1.2. Research Progress on Colored 3D Printing Materials

In the field of colored 3D printing materials, existing studies have explored coloring methods and color presentation effects. Rupp et al. designed responsive structurally colored 3D printing materials based on core–shell particle structures [18], and their concept of physical color generation provided a different technical pathway for this study to achieve coloration through inorganic pigments. Meng et al., inspired by the selective light scattering phenomenon of disordered structures on blueberry surfaces, employed Mie scattering to induce structural colors in polylactic acid (PLA) particles and produced fused deposition modeling (FDM) filaments with uniform diameter and tunable color via melt extrusion [19]. Their process route of “pigment and powder blending, slurry extrusion, and curing and color development” inspired the basic workflow adopted in this study for preparing colored slurries and printing, and also provided ideas for optimizing coloring effects from the perspective of particle size distribution. Kissel et al. established a spectral Monte Carlo path-tracing rendering model based on the intrinsic optical parameters of materials, achieving high-fidelity prediction of 3D-printed object colors (color difference ΔE < 2.0) [20]. This quantitative color evaluation method can be directly transferred to the color difference analysis in this study to guide the fine-tuning of pigment dosage. Zhang et al. developed printable structurally colored inks based on cholesteric cellulose liquid crystals and achieved self-supporting structural color printing by optimizing rheological properties with gelatin [21]. Their approach of adjusting ink rheology via additives to suit printing processes inspired the research idea of improving slurry rheological behavior through mix proportion adjustment in this study, and also demonstrated that rheological regulation plays a key role in colored printing.

The above studies indicate that research on colored 3D printing materials has attracted increasing attention, with their application value continuously expanding in both functional and customized directions. However, research on inorganic cementitious material systems, especially solid waste slag-based materials, remains relatively limited.

#### 1.1.3. Research Progress on Colored Cement-Based Materials

Currently, there is a lack of systematic research specifically targeting colored slag-based materials. However, in the closely related field of colored cement-based materials, scholars have conducted relatively systematic explorations in areas such as color matching regulation, production process optimization, and mechanical properties, and the relevant findings provide important theoretical and methodological references for this study. Xia et al. established an inorganic pigment color matching model based on magnesium phosphate cement, achieving color compatibility between repair materials and existing concrete structures [22], and their linear color mixing theory provided a methodological reference for the quantitative color characterization of the colored slag system in this study. Russo et al. confirmed that inorganic pigments can achieve effective coloration without significantly sacrificing the mechanical properties of concrete [23], supporting the hypothesis that “coloration and strength can be synergistically optimized” in this study. Yıldızel revealed the differentiated effects of different colored pigments on the performance of self-compacting concrete [24], providing an experimental design basis for the systematic comparison of red, yellow, and blue pigments in this study. El Nemr et al. quantified the coupled effects of pigment dosage and water-to-binder ratio on the mechanical properties of colored mortar [25], offering a theoretical explanation for the rationality of adjusting the water-to-solid ratio after pigment incorporation in this study.

In summary, while existing research has made certain progress in the field of colored cement-based materials, studies on colored 3D printing materials specifically for solid waste slag systems are still lacking. Against this background, this study uses two types of industrial solid waste, blast furnace slag and desulfurized gypsum, as the main raw materials to systematically evaluate the coloring behavior, rheological properties, mechanical properties, and printability of colored slag–desulfurized gypsum composite materials, aiming to provide a basis for the high-value utilization of solid waste resources and the development of colored 3D printing materials.

#### 1.1.4. Summary

Based on the above literature review, the following research gaps can be identified: (1) Existing studies on slag-based 3D printing materials mainly focus on improving mechanical properties and printability, while the combined optimization of color appearance and material performance has been largely neglected. (2) The color stability and pigmentation uniformity of iron oxide pigments in slag-based matrices have not been systematically evaluated. (3) The influence of key printing parameters, such as water temperature, on the setting time of colored slag-based materials has rarely been investigated.

### 1.2. Research Objectives and Significance

Color 3D printing can achieve realistic color reproduction for 3D objects [26,27]. This study uses blast furnace slag as the primary material and develops colored SB-3DPM suitable for extrusion-based processes. These materials allow coloring and model fabrication to occur at the same time, which shortens the production cycle. Traditional surface coloring often leads to pigment peeling, cracking, and detachment [28]. In this study, pigments are bonded to the slag-based matrix through chemical interactions, forming an integrated composite material. This approach effectively avoids these problems. In addition, introducing the coloring step at an earlier stage reduces air pollution caused by volatilization during paint spraying. This approach is consistent with the principles of green manufacturing.

The development of colored SB-3DPM can transform the traditional low-value-added reuse of slag waste and promote high-quality sustainable development. Enriched visual expression of SB-3DPM helps improve public acceptance and strengthens market competitiveness [29]. Studies show that the AM market may reach $2 trillion [30]. Colored SB-3DPM offers significant cost advantages and unique environmental benefits. They have the potential to create new growth points in the green economy and to secure a position in the sustainable 3D printing market.

## 2. Design and Preparation of Colored SB-3DPM

### 2.1. Selecting Raw Materials

#### 2.1.1. Slag-Based Cementitious System

This study follows a sustainability-oriented approach and aims to maximize environmental benefits. As industrial by-products, blast furnace slag and desulfurized gypsum were selected as the main raw materials [31,32]. The mix design is based on the formulation proposed by Li et al. [17]. In that study, an L_9_(3^3^) orthogonal experiment was conducted with blast furnace slag as the primary cementitious component (dosage: 60 g, 75 g, 90 g), desulfurized gypsum as the supplementary cementitious material (dosage: 30 g, 45 g, 60 g), and water as the admixture (34 g, 37 g, 40 g), to systematically investigate the effects of each factor on the compressive strength of the material. The specimens were cured under natural indoor conditions for 45 days before compressive strength testing, with three parallel specimens per group averaged and the failure modes recorded. The experimental results showed that both the mixture with 75 g slag, 45 g gypsum, and 40 g water and the mixture with 90 g slag, 30 g gypsum, and 40 g water achieved a compressive load exceeding 100,000 N, reaching the maximum capacity of the testing machine. However, the specimens with 90 g slag, 30 g gypsum, and 40 g water exhibited severe edge damage, showing obvious brittle failure characteristics and poor homogeneity; by contrast, the specimens with 75 g slag, 45 g gypsum, and 40 g water exhibited significantly less edge damage while withstanding the maximum load, with a more uniform failure mode, indicating superior structural integrity and homogeneity under this mix proportion. Based on the comprehensive evaluation of the above mechanical properties and failure modes, Li et al. [17] recommended 75 g slag, 45 g gypsum, and 40 g water (mass ratio 15:9:8) as the optimal mix proportion. The reference composition includes 75 g of slag, 45 g of gypsum, and 40 g of water, with a mass ratio of 15:9:8. Previous studies have shown that this proportion provides high compressive strength. This study further optimizes the reference mix. It maintains adequate printability of the material. At the same time, functional colored materials are introduced. The aim is to develop colored SB-3DPM with both decorative properties and load-bearing capacity. The overall performance of the prepared specimens is then evaluated.

#### 2.1.2. Selection and Analysis of the Pigment System

In this study, the selection of coloring admixtures follows a principle of sustainability first. Eco-friendly iron oxide pigments were chosen [33]. These pigments are optimized in production for particle size and impurity content, which prevents nozzle clogging during printing. Iron oxide pigments are highly stable at high temperatures [34]. This ensures that their color remains unchanged under the high-temperature conditions of 3D printing. Furthermore, discarded iron oxide pigments naturally degrade back into iron oxides. Unlike persistent organic pigments, they do not cause long-term pollution [35]. In terms of pigment dosage determination, this study conducted preliminary experiments to test the color difference values of specimens with iron oxide pigment dosages of 1 g, 2 g, 4 g, 6 g, 8 g, 10 g, 12 g, and 14 g. The experimental results showed that when the pigment dosage was below 2 g, the color difference change was not significant, indicating poor coloring effectiveness; when the dosage exceeded 10 g, the increase in color difference slowed markedly, suggesting that the color approached saturation. Considering coloring efficiency, three dosage levels of 2 g, 6 g, and 10 g were selected as experimental variables, representing low, medium, and high dosage levels, respectively. On the basis of the reference mix proportion (62.5 g slag, 37.5 g gypsum, and 33.3 g water), the above three dosages of iron oxide pigment powder were added. Since the addition of pigment powder increased the total mass of the powder, and to ensure consistent flowability and extrudability of the slurry and avoid clogging during mixing, the water content was uniformly increased from 33.3 g to 36 g (water-to-solid ratio adjusted from 0.333 to approximately 0.343) based on the flowability test results in the preliminary experiments, so as to ensure that the initial flowability of the slurries under different dosages remained essentially consistent, facilitating subsequent comparative evaluation of printing performance. Although the slight increase in water-to-solid ratio may lead to a minor reduction in compressive load, this adjustment was necessary to ensure consistent printability across all groups, and the effect was considered acceptable given that the same ratio was applied uniformly, ensuring a fair comparison.

### 2.2. Color Mixing Experiment

#### 2.2.1. Optimization of Particle Size Distribution for SB-3DPM Using the Andreasen Model

When preparing SB-3DPM, differences in particle size between slag powder and desulfurized gypsum powder can affect slurry flowability and packing density. In this study, the Andreasen model [36] was applied to determine the ideal curve of particle size distribution for the mixture of slag and gypsum powders. In powder engineering, the Andreasen equation is widely used to evaluate and optimize the packing density of particulate systems. When the particle size distribution approaches the theoretical densely packed state, both slurry compactness and material mechanical properties can be improved [37]. Equation (1) is expressed as follows:

(1) $$P_{target}(D)={\left(\frac{D}{D_{max}}\right)}^{q}$$

where D represents the particle size, and D_max_ represents the size of the largest particle in the system. P_target_(D) represents the cumulative mass percentage of particles smaller than D. The exponent q denotes the distribution modulus, which determines the shape of the curve. Our measurements showed that slag powder had a particle size range of 10–50 μm, while desulfurized gypsum powder ranged from 30 to 60 μm. The maximum particle size of gypsum was taken as 60 μm (D_max_ = 60 μm). To ensure adequate strength of the mixture, the distribution modulus was set to 4.5 (q = 4.5). A previous study [38] has shown that increasing the proportion of coarse particles can effectively enhance the mechanical properties of materials. Based on this, the present study adopted a relatively high q value of 4.5 to obtain a grading curve with a higher proportion of coarse particles, thereby constructing a coarse particle skeleton structure, which is conducive to improving the support capacity of the material during printing to prevent collapse, and also contributes to enhancing the structural strength of the hardened printed models. The particle size distribution was measured using a QL-2077 laser particle size analyzer (Qunlong, Xiamen, China). Dry measurements were performed on a total of 1000 g of mixed powder, consisting of 625 g of slag and 375 g of desulfurized gypsum. Representative particle sizes covering both powders were selected and applied to the formula. The calculation results are presented in Table 1.

To evaluate the reliability and repeatability of the measured particle size distribution data, three independent repeated laser particle size measurements (n = 3) were performed on the same mixed powder sample. The cumulative mass percentages at each particle size point were averaged, and the standard deviation (SD) was calculated. The calculated standard deviations at all representative particle size points were ≤1.1%, indicating that the instrument measurement fluctuation was minimal and the data exhibited good repeatability. As shown in Table 1, the deviations between the measured values and the Andreasen theoretical values ranged from –7.34% to +6.43%, with the measured value at the 10 μm particle size point being approximately 7.34% lower than the theoretical value. Drawing on the research approach of particle size distribution optimization in cement-based materials [39], when the particle size distribution of cementitious materials more closely meets the requirements of the densely packed state, the density of cement paste increases and compressive strength is significantly improved. This particle size distribution optimization principle is also applicable to the slag–desulfurized gypsum composite powder system in this study, i.e., improving packing density can enhance the mechanical properties of the hardened material. The deviations between the measured curve and the theoretical curve at each particle size point in this study were limited, with only the deviation at 10 μm being relatively pronounced. In powder dense packing theory, deviations between actual grading and the theoretical curve within the range of ±5% to ±10% are generally considered acceptable. The deviation at 10 μm in this study was approximately 7.34%, which lies at the boundary of this range, indicating that while this mix proportion does not seriously deviate from the theoretical requirements, there remains room for optimization.

**Figure 1:**
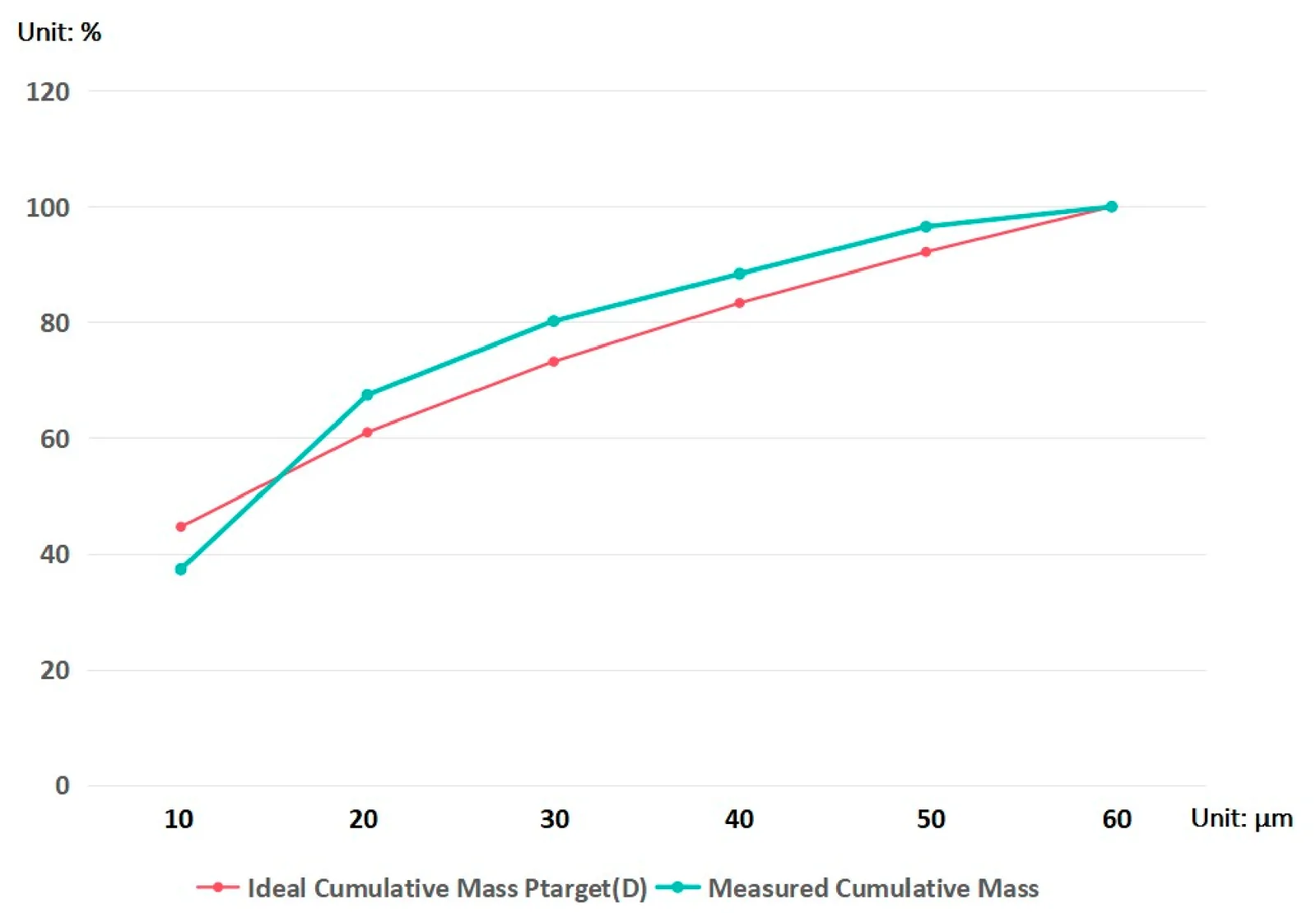
Comparison of measured and target cumulative particle size distributions.

The comparison in Figure 1 shows that, for particle sizes of 20 μm and above, the cumulative mass of the mixture exceeds the ideal values. At 10 μm, it falls below the ideal curve, indicating a shortage of fine particles smaller than 10 μm. This is caused by the coarse desulfurized gypsum powder. For particle sizes above 30 μm, the mixture contains both slag and gypsum, resulting in a cumulative mass higher than the ideal. Below 20 μm, the mixture consists of slag only. The limited amount of fine slag particles prevents the cumulative mass from reaching the ideal value.

To address the issue above, in systems without pigment powder, the particle size distribution of the mixture can be optimized by increasing the proportion of slag powder smaller than 20 μm or by reducing the proportion of desulfurized gypsum powder. In systems containing pigment powder, the fine end of the distribution can be enhanced by adding iron oxide pigment, which has a particle size below 10 μm. This increases the cumulative mass of fine particles and helps approach the ideal curve. If iron oxide pigment is added while maintaining a distribution close to the ideal, coarse slag can be increased appropriately to adjust the overall particle size distribution.

#### 2.2.2. Selection of Measurement Points and Original L*, a*, b* Color Values

**Figure 2:**
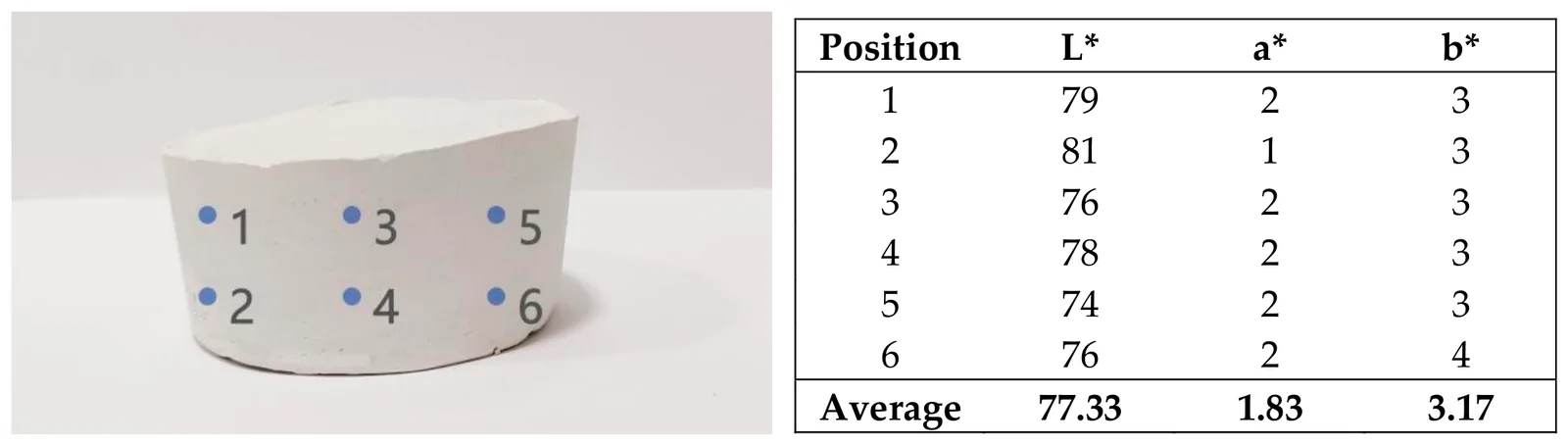
Measurement points and average values of L*, a*, and b*.

For the experiment, original specimens were prepared with 62.5 g of slag, 37.5 g of desulfurized gypsum, and 33.3 g of water. The surface color of each specimen was measured at six points using a handheld DS-200 colorimeter (Caipu, Shantou, China) with a 3 mm aperture. In the CIELAB color space, L* represents lightness (0 = black, 100 = white), a* represents the red–green axis (positive values indicate red, negative values indicate green), and b* represents the yellow–blue axis (positive values indicate yellow, negative values indicate blue). These three parameters provide a quantitative description of surface color and serve as the basis for calculating the color difference ΔE between specimens. The values of L*, a*, and b* were recorded at each point (Figure 2). The average of these six measurements was calculated to determine the reference color of the sample, as shown in Figure 2.

#### 2.2.3. Preparation of Colored Samples and Changes in Color Difference

Three types of iron oxide pigments were used in this study: Pigment Red 101 (Fe_2_O_3_), Pigment Yellow 42 (FeOOH·H_2_O), and Pigment Blue 27 (Fe_4_[Fe(CN)_6_]_3_). Starting with the original mix of 62.5 g slag, 37.5 g desulfurized gypsum, and 33.3 g water, different amounts of iron oxide pigment (2 g, 6 g, and 10 g) were added. Because the increased total powder mass reduced the relative water content, the water amount was increased to 36 g to prevent agglomeration during mixing and to ensure that the initial flowability of the slurries under different dosages remained essentially consistent. For color measurements, three parallel specimens were prepared for each mix proportion. Six measurement points were selected on the surface of each specimen (as shown in Figure 2), yielding a total of 18 measurement points (n = 18). The average value was taken as the final colorimetric value for each mix proportion, and the standard deviation (SD) was calculated. All color data are presented as mean ± SD, as shown in Table 2, Table 3 and Table 4.

The CIELAB color difference formula [40] was used to quantify the color change induced by different pigment dosages. The color difference (ΔE) between the specimen’s current color and its original (uncolored) state was calculated using Equation (2):

(2) $$\Delta_{E}=\sqrt{{\left({L^{*}}_{1}-{L^{*}}_{2}\right)}^{2}+{\left({a^{*}}_{1}-{a^{*}}_{2}\right)}^{2}+{\left({b^{*}}_{1}-{b^{*}}_{2}\right)}^{2}}$$

where L*_1_, a*_1_, and b*_1_ represent the average values after adding the pigment powders, while L*_2_, a*_2_, and b*_2_ represent the average values of the original specimen. The calculation results are shown in Table 5.

**Figure 3:**
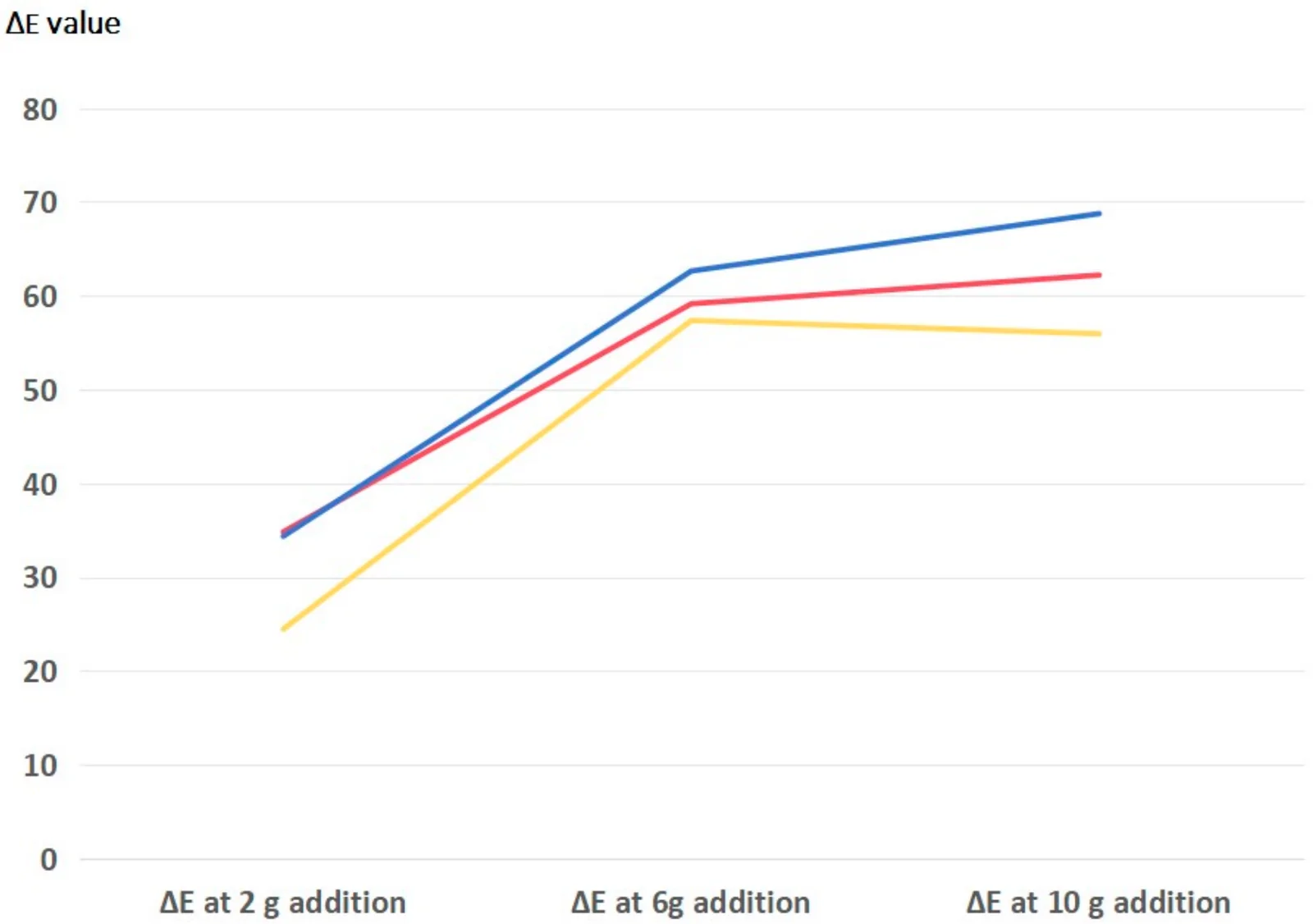
Line graph of color difference for three color specimens with different pigment additions.

From the trend shown in Figure 3, when the pigment amount increased from 2 g to 6 g, the color of the specimens changed significantly, and ΔE grew. However, when the pigment increased from 6 g to 10 g, the color change was smaller, and the ΔE became more similar. Notably, the color difference in the specimen with 10 g of Pigment Yellow 42 was smaller than in the specimen with 6 g of that pigment. This indicates that there is an obvious law of “diminishing marginal effect” in the coloring process: when the pigment dosage is low, pigment particles gradually fill the surface and internal voids of the matrix, resulting in high coloring efficiency and rapid increase in color difference; when the pigment concentration approaches the saturated adsorption capacity of the matrix surface, the excess pigment particles begin to aggregate or exist in a multi-layer stacking form, contributing significantly less to color development. This indicates that the pigment coloring in the composite material of slag and desulfurized gypsum has a saturation limit. After a certain concentration, adding more pigment has little effect on further color changes.

According to Table 5, Pigment Yellow 42 shows the largest increase in ΔE when the dosage increases from 2 g to 6 g. The increase is about 134.6%. ΔE reaches a maximum at 6 g and then decreases slightly at 10 g. This decrease may be related to the relatively poor dispersibility of Pigment Yellow 42 in the matrix: when the dosage exceeds 6 g, the probability of pigment particle agglomeration increases, causing the actual color-developing area in the matrix to not increase but decrease, resulting in a slight decline in color difference. Pigment Blue 27 exhibits the second-largest increase, at about 82.3%. However, the rate of increase slows significantly after 6 g. This may be due to the fact that the tinting strength of Pigment Blue 27 lies between that of yellow and red pigments, and its saturation concentration point is relatively well-matched with the adsorption capacity of the matrix, thus no obvious decline is observed. Pigment Red 101 exhibits a smaller increase than the other two pigments, at about 69.7%. ΔE approaches saturation after 6 g. The coloring efficiency of Pigment Red 101 in this matrix is relatively moderate, and its coloring process tends to follow a gradual saturation pattern without obvious over-saturation decline, which may be related to its particle surface characteristics and compatibility with the matrix.

From an economic perspective, the pigment dosage can be set at about 6 g. This level provides the best cost–performance balance. It achieves a near-saturated color while minimizing pigment consumption during material preparation. From the perspective of economic feasibility, based on the relationship between color difference increase and pigment dosage, the maximum pigment dosage can be controlled at approximately 6 g in future applications, which represents the optimal cost–performance threshold, achieving near-saturated color effects at the lowest cost and saving iron oxide pigment costs during material preparation.

### 2.3. Color Uniformity

This study evaluated the uniformity of pigment distribution in specimens colored with iron oxide pigments. Three specimens were prepared with 6 g of red, yellow, and blue pigments, respectively. For each specimen, three observation points were randomly selected on the surface and examined under an optical microscope at 40× magnification. Digital images were analyzed using ImageJ to quantify color characteristics. The mean intensity of each channel was used to assess color tendency and the uniformity of pigment distribution. Each pixel in the image consists of intensity values from the red, green, and blue channels. If one channel exhibited consistently higher intensity while the other two remained lower, the specimen tended toward the base color represented by that channel. Fluctuations in channel intensity indicated non-uniform color.

As shown in Table 6, the three images of the red specimen had significantly higher intensity in the red channel compared with the green and blue channels, indicating a clear red bias. The average intensity of the red channel was 143.04, the green channel was 212.14, and the blue channel was 154.59. The standard deviation of the intensity values in the red channel across the three captured images was 3.49, which is much less than 10% of its average value, indicating that the intensity fluctuation within the images was small. In the red channel images, no obvious bright or dark spots were observed, except in pore regions, and the intensity was evenly distributed. Combined with the small standard deviation of the red channel, this quantitative analysis result intuitively demonstrates that, under the experimental conditions of this study, Pigment Red 101 was uniformly distributed in the slag–desulfurized gypsum composite, without excessive pigment concentration or agglomeration. These observations demonstrate that, under the experimental conditions, Pigment Red 101 was uniformly distributed in the slag–desulfurized gypsum composite. No pigment agglomeration or local overconcentration occurred. This is because the dosage of Pigment Red 101 was within a reasonable range of the matrix dispersion capacity, enabling relatively uniform distribution during slurry mixing and molding, without pigment agglomeration caused by over-saturation.

As shown in Table 7, the intensities of the red and green channels were higher than those of the blue channel, and the intensities of the red and green channels were similar. The average intensity of the red channel was 193.68, the green channel was 179.06, and the blue channel was 147.70. The difference between the red and green channels was approximately 15 units, which is much smaller than the difference between the red and blue channels (approximately 46 units). In the additive RGB color model, yellow results from a combination of red and green. This indicates a clear yellow bias in the specimen. Across the three captured images, the standard deviation of the intensity values was 11.80 for the red channel, 11.86 for the green channel, and 10.26 for the blue channel, all within a reasonable fluctuation range. Except for the pore regions, the images showed no significant bright or dark spots, and the intensity distribution was uniform. The experimental results indicate that, under the experimental conditions used in this study, Pigment Yellow 42 exhibited good dispersion uniformity in the slag–desulfurized gypsum composite, without pigment agglomeration or excessive concentration. This indicates that the dosage of Pigment Yellow 42 did not exceed the upper limit of the matrix dispersion capacity, and the pigment could achieve relatively uniform distribution during slurry mixing and molding. These results indicate that, under the experimental conditions used in this study, Pigment Yellow 42 was evenly dispersed in the slag–desulfurized gypsum composite. No pigment agglomeration or local overconcentration occurred.

As shown in Table 8, the selected specimen images exhibited higher intensity in the blue and green channels, while the intensity of the red channel was lower. The intensities of blue and green channels were also similar. The average intensity of the blue channel was 189.91, the green channel was 188.44, and the red channel was 145.68. The difference between the blue and green channels was only 1.47 units, which is much smaller than the difference between the red and blue channels (approximately 44 units). In the additive RGB color model, when the blue and green channels were similar, and the red channel was lower, the color appears cyan-leaning. This indicates a clear blue bias in the specimen. Across the three captured images, the standard deviation of the intensity values was 1.48 for the red channel, 2.00 for the green channel, and 1.77 for the blue channel, all within a small fluctuation range, indicating uniform intensity distribution. Except for the pore regions, the images exhibited no significant bright or dark spots, and the intensity distribution was uniform. The experimental data confirmed that, under the experimental conditions set in this study, Pigment Blue 27 could be uniformly dispersed in the slag–desulfurized gypsum composite, without pigment aggregation or local overconcentration. This indicates that the dosage of Pigment Blue 27 was within a reasonable range of the matrix dispersion capacity, and the pigment could achieve uniform distribution during slurry mixing and molding. These results confirmed that, under the experimental conditions used in this study, Pigment Blue 27 was evenly dispersed in the slag–desulfurized gypsum composite. No pigment agglomeration or local overconcentration occurred.

## 3. Print Performance of Materials

### 3.1. Effect of Temperature on Setting Time

In the field of AM, printing temperature is critical in a material’s flow behavior, hardening rate, and the performance of the final product [41]. Understanding how temperature affects the hardening reaction enables better control of 3D-printed model quality. This study set water temperature at different levels to investigate how different proportions of iron oxide pigments influenced the hardening rate of the slag–desulfurized gypsum composite. It also analyzed the combined effect of water temperature and pigment addition on the hardening time.

In this study, the hardening time was determined using an SC-145 mortar fluidity tester (Tongli Jiudu, Cangzhou, China) based on the penetration resistance method. The lever was pressed vertically downward, and the test needle was inserted into the mixture within 5 s. The initial measurement was recorded and repeated every minute. When the penetration resistance reached 0.3 MPa, the measurement interval was shortened to every 30 s until the resistance reached 0.6 MPa. The time from the start of mixing to this point was recorded as the hardening time. The temperature variation in the mixture was monitored using an infrared thermometer aimed at the center of the mixture, with the temperature recorded every 30 s.

**Figure 4:**
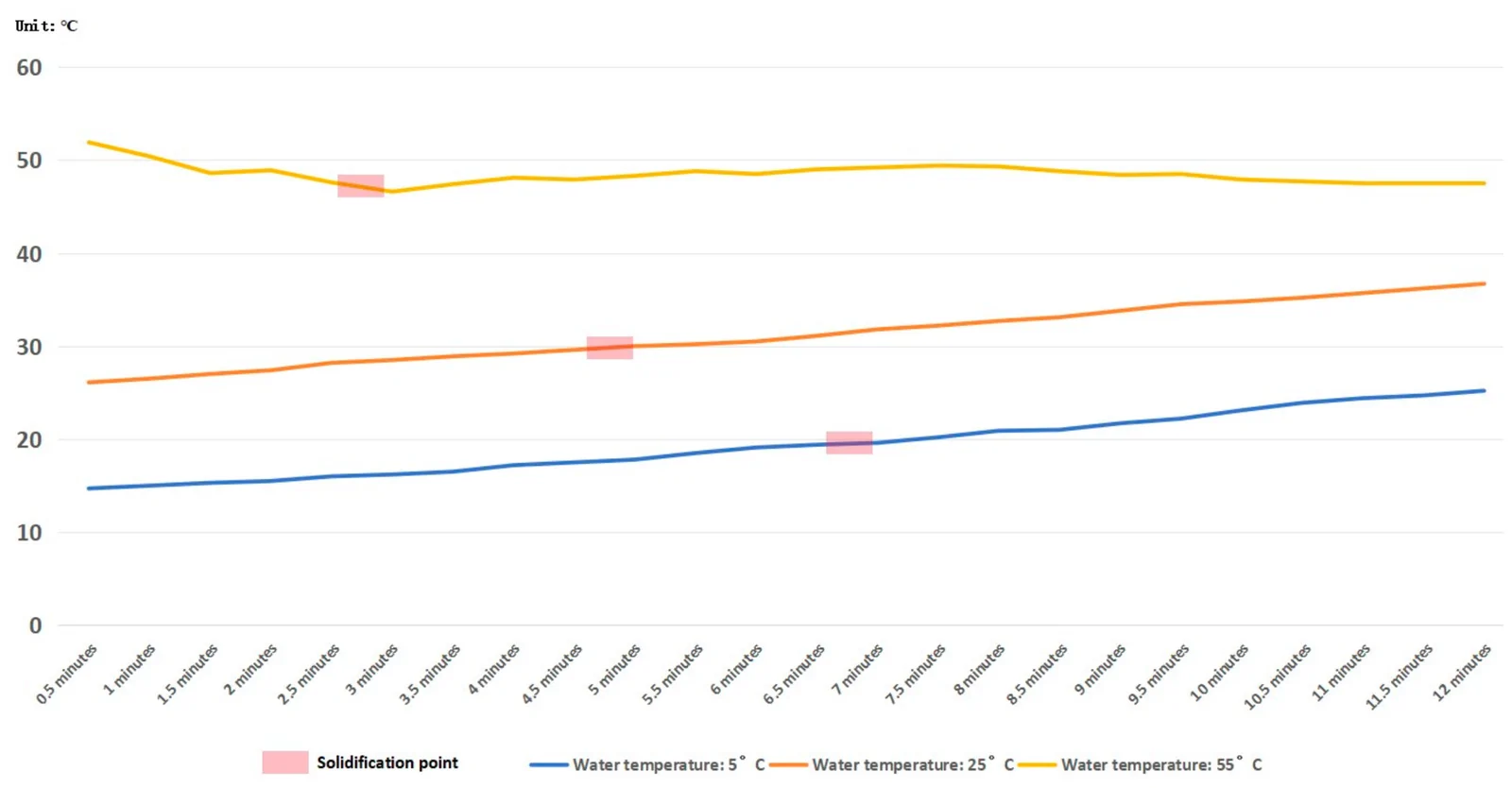
Effect of water temperature on the setting reaction of the base formulation.

The base formulation without pigments was tested first. The mixture consisted of 62.5 g slag, 37.5 g desulfurized gypsum, and 33.3 g water. After thorough stirring for 30 s, the hardening reaction was monitored at water temperatures of 5 °C, 25 °C, and 55 °C. The results are summarized in Figure 4.

The experiment showed that the hardening reaction of the mixture (slag, desulfurized gypsum, and water) was obviously accompanied by heat release. At a water temperature of 5 °C, the setting time was delayed. At 55 °C, the setting time was significantly shortened.

To further study how iron oxide pigments affect the hardening reaction of the slag–desulfurized gypsum composite, and to determine whether water temperature still influences the hardening speed when pigments are present, we fixed the mass ratio of slag to desulfurized gypsum at 62.5:37.5 and kept the water content at 36 g. Three pigments were tested: Pigment Red 101, Pigment Yellow 42, and Pigment Blue 27. Each pigment was added at three levels: 2 g, 6 g, and 10 g. Three water temperatures were also tested: 5 °C, 25 °C, and 55 °C. The experiments were designed and conducted based on an L_9_(3^4^) orthogonal array, and nine experimental runs were conducted, as shown in Table 9. The setting time of the mixture was measured using an SC-145 mortar fluidity tester. The operator pressed the rod vertically and forced the test needle into the mixture within 5 s. The first reading was recorded. The test was then repeated every 15 s until the resistance reached 20 MPa. The time required to reach this value was taken as the approximate setting time.

Based on the data in Table 9, a range analysis was conducted. The average setting time for each factor at each level was calculated (Table 10). Water temperature had the greatest influence on the setting time, with a range of 255 s. The second most influential factor was the pigment dosage, with a range of 15 s. Pigment type had the least effect, with a range of only 5 s. When the water temperature increased from 5 °C to 55 °C, the average setting time decreased from 380 s to 125 s, a reduction of 67.1%, indicating that temperature plays a significant regulatory role in the hydration reaction rate of the slag–desulfurized gypsum system. When the pigment dosage increased from 2 g to 10 g, the average setting time decreased from 255 s to 240 s, a reduction of approximately 5.9%, indicating a relatively mild accelerating effect. The effect of pigment type on setting time was minimal (red: 250 s, yellow: 245 s, blue: 245 s), and the differences among the three were negligible within the experimental error range.

In summary, water temperature can either accelerate or delay the setting of the colored slag–desulfurized gypsum composite. In 3D printing, adjusting the temperatures of the nozzle and heated bed accelerated hardening. This gave the extruded material stronger support, allowed it to stack and hold its shape, and thus prevented model collapse. This is because the hydration reaction of slag is exothermic; increasing the temperature accelerates the dissolution of ions and the formation rate of hydration products, thereby shortening the setting time. Conversely, lowering the temperature during material transport prolonged the setting time. This maintained optimal fluidity and made extrusion easier. At a low temperature of 5 °C, the setting time was extended to 380 s, providing a sufficient operating window for pumping and extrusion of the material. Moreover, increasing pigment dosage slightly accelerated the composite’s hardening. This may be due to the large specific surface area of the pigment fine powder, whose incorporation increases the solid-phase interface in the system, providing more nucleation sites for the precipitation of hydration products, thereby exerting a slight promoting effect on the early hydration reaction. Therefore, when printing models with more vivid colors, the minor acceleration of the setting caused by higher pigment content should be considered.

### 3.2. Shear-Thinning Behavior and the Feasibility of 3D Printing

**Figure 5:**
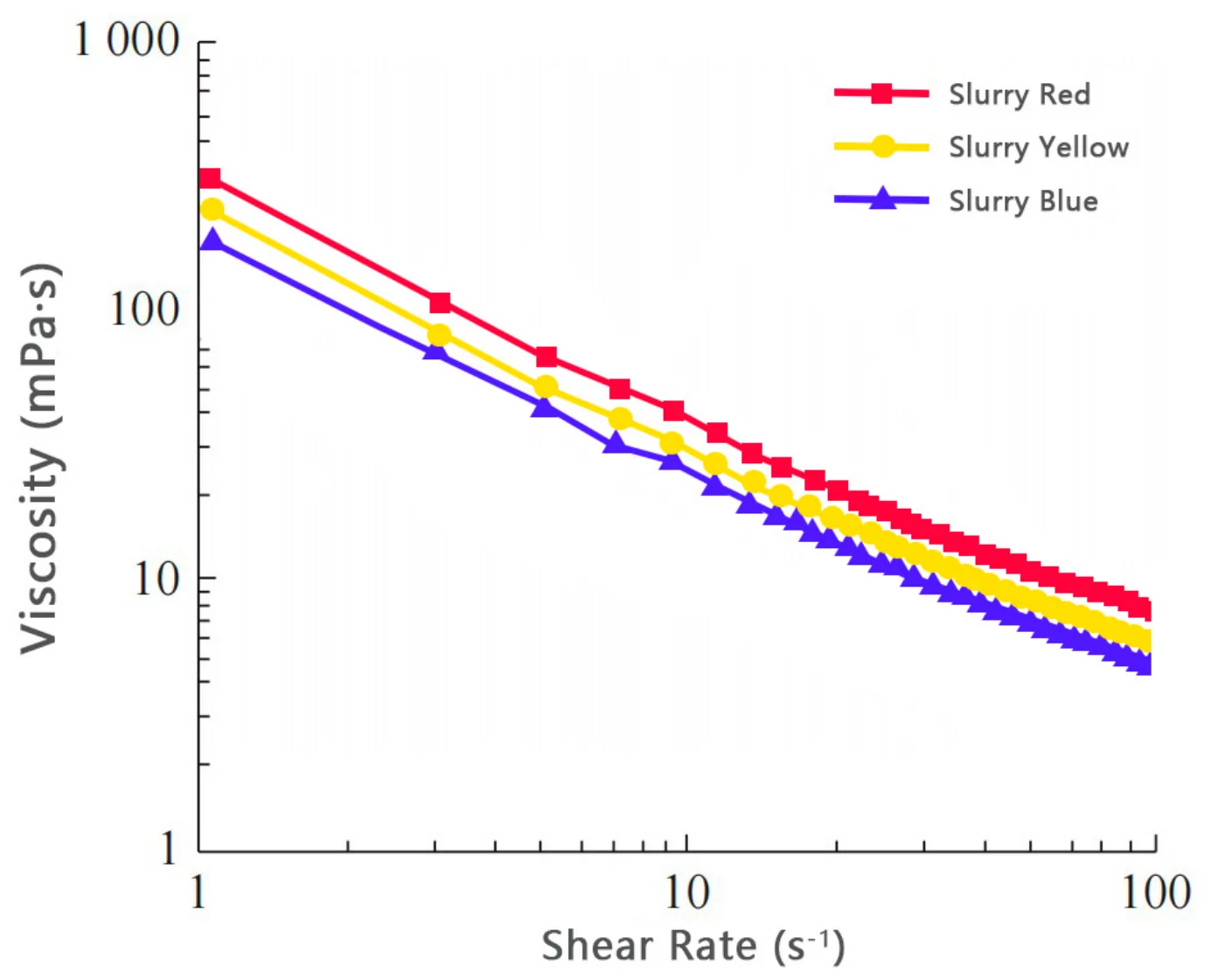
Rheological properties of slurries with different pigments.

The slurry’s rheological behavior is a key factor in extrusion-based 3D printing. It determines print success. The colored slag–desulfurized gypsum composite must flow smoothly through the nozzle while retaining enough plasticity to prevent collapse. The rheological behavior of the colored slag–desulfurized gypsum composite can be described by the Herschel–Bulkley model [42], which can simultaneously characterize yield stress and shear-thinning behavior, matching the rheological requirements of 3D printing slurries during extrusion and deposition. In this study, the rheological parameters were measured using a rotational rheometer. The slurry was placed between parallel plates, and the shear rate was increased stepwise. The shear stress and apparent viscosity were recorded at each shear rate. The flow curve was then fitted using the Herschel–Bulkley model to obtain the yield stress, consistency coefficient, and flow index. This study examined the rheology of the colored slag–desulfurized gypsum composite under different shear rates. Viscosity changes in the three pigment slurries were recorded, as shown in Figure 5. As the shear rate increased, all slurries exhibited shear-thinning behavior, and viscosity decreased. When the shear rate exceeded 10^−1^ s^−1^, viscosities dropped below 50 Pa·s, allowing smooth extrusion from the nozzle. The shear-thinning behavior is beneficial to the printing process: under the high shear rate within the nozzle, the slurry viscosity decreases, reducing flow resistance and facilitating extrusion; after extrusion, the shear rate drops sharply, and the viscosity recovers quickly, which helps maintain the printed shape and prevents collapse.

The rheological behavior of the colored slag–desulfurized gypsum composite follows the Herschel–Bulkley model [43], expressed in Equations (3) and (4):

(3) $$\tau \;=\;\tau_{0}\;+{K\dot{\gamma }}^{n}$$

(4) $$\eta \;=\;\frac{\tau_{0}}{\dot{\gamma }}+{K(\dot{\gamma })}^{n-1}$$

where τ represents the shear stress, τ_0_ represents the yield stress, K represents the consistency coefficient, γ represents the shear rate, n represents the flow index, and η represents viscosity. When 0 < n < 1, the slurry behaves as a yield-pseudoplastic fluid and exhibits shear-thinning. Among these parameters, the yield stress τ_0_ reflects the material’s resistance to deformation under static conditions; a higher τ_0_ indicates stronger load-bearing capacity between deposited layers after extrusion. A smaller flow index n indicates a more pronounced shear-thinning effect and lower flow resistance during extrusion. For this experiment, the yellow slurry, whose values remained intermediate among the three trends, was selected for analysis. The measured yield stress was τ_0_ = 201 Pa, the consistency coefficient K = 19.8 Pa·s^n^, and the flow index n = 0.61. The calculated result of n = 0.61 satisfies 0 < n < 1, indicating that the slurry exhibits typical shear-thinning characteristics and meets the rheological requirements for printability in extrusion-based 3D printing [44].

### 3.3. Extrusion Printing and Performance Evaluation

**Figure 6:**
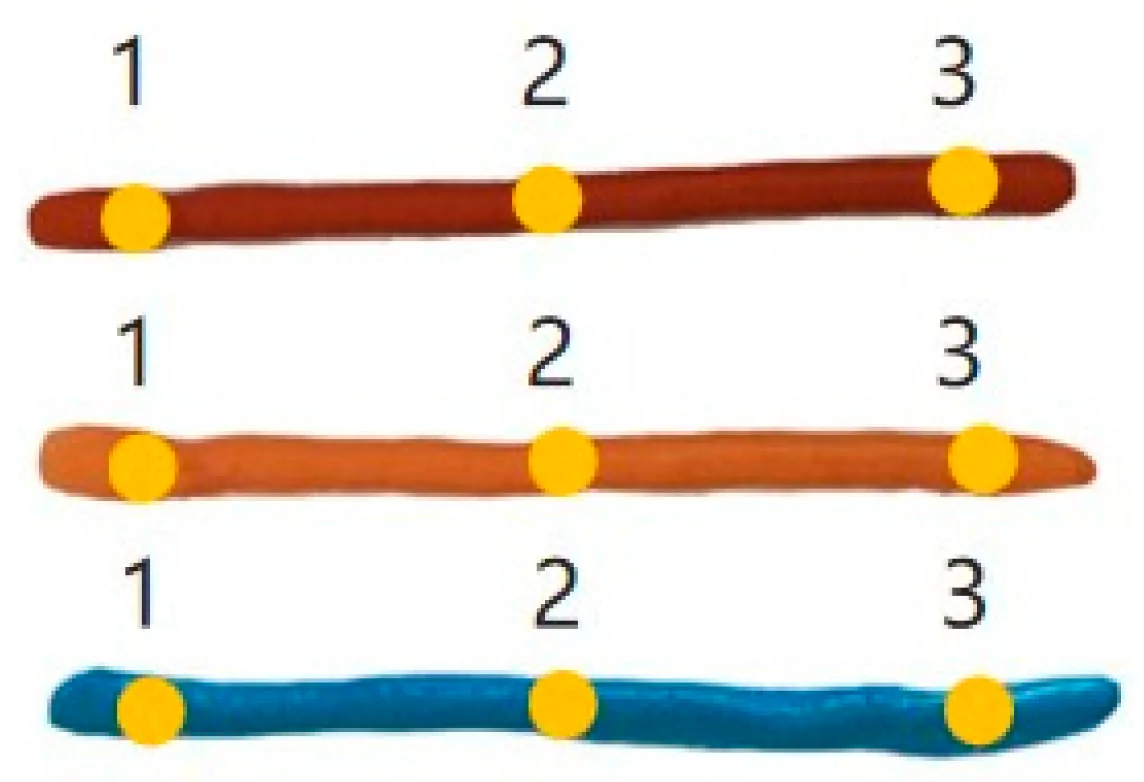
Schematic diagram of the measurement point locations on the specimens.

Extrusion tests were conducted using a CERAMBOT EAZAO desktop 3D printer (Chuangxingyuan, Shenzhen, China). The nozzle diameter was 5 mm, and the printing speed was 10 mm/s. The nozzle height was set at approximately 5 mm above the printing platform, and the printing path was designed as straight lines of 80 mm in length. The extrusion pressure was adjusted in real time in the range of 0.2–0.4 MPa according to the slurry flowability to ensure continuous and uniform extrusion from the nozzle. During printing, the ambient temperature was maintained at 20–25 °C, with a relative humidity of 50–60%. After each straight-line specimen was printed, it was allowed to stand for 2 min for initial curing, and then the strip-shaped specimens were carefully removed from the printing platform and continuously cured under the same environmental conditions. For each pigment formulation, three specimens were tested, with three measurement points taken per specimen (as shown in Figure 6). The extruded width, extruded height, and post-curing height of the strip-shaped specimens were recorded, and compressive strength tests were performed. The results were averaged. The printing speed of 10 mm/s and nozzle height of 5 mm were determined based on the optimal results for obtaining continuous and uniform extruded lines in preliminary experiments; the extrusion pressure was finely adjusted in real time according to the slurry flowability to ensure stable extrusion. Linear specimens of 80 mm length were printed using the slag–desulfurized gypsum composite with three different pigment formulations. Extruded filament dimensions were recorded, and compressive strength tests were conducted. The results are presented in Table 11.

The specimens showed stable printability. Extruded lines were continuous and uniform. Volume shrinkage after curing was minimal. At a printing speed of 10 mm/s and extrusion pressure of 0.2–0.4 MPa, the average extrusion width of the three colored specimens was 6.45 mm (range: 0.16 mm, relative standard deviation: approximately 1.1%), and the average extrusion height was 5.86 mm (range: 0.05 mm, relative standard deviation: approximately 0.4%). The fluctuations in both dimensional parameters were small. These results indicate that the colored slag–desulfurized gypsum composite maintained excellent structural integrity and dimensional stability. The compressive strengths of the three colored specimens were 20.03 N, 19.93 N, and 19.13 N, respectively, with an average of 19.70 N and a range of only 0.90 N, indicating a low degree of dispersion, which further confirms the good structural uniformity and processing stability of the material.

During printing, the nozzle remained free of clogging or particle agglomeration. All three composites, each with different pigment contents, were continuously and uniformly extruded. No breakage or intermittent flow occurred, and the extruded strands showed no visible voids on the surface. This further confirms that the pigment was uniformly dispersed in the material without agglomeration, which is consistent with the conclusions on pigment dispersion uniformity drawn from the previous color characterization. It was also observed that, although the nozzle diameter was 5 mm, the extruded strands were larger than the nozzle opening. The average extrusion width of 6.45 mm was approximately 1.3 times the nozzle diameter. This indicates that the material exhibits clear rheological behavior. Under strong shear during extrusion, its viscosity decreased. Once extruded, the pressure was released, and the microstructure elastically recovered, causing the strand diameter to expand beyond the nozzle size.

The study also measured the average height of the specimens before and after curing. All three groups exhibited slight height shrinkage (1.02%, 0.51%, and 0.34%, respectively). The overall shrinkage was below 1.1%, indicating good dimensional stability and that the single-strand specimens maintained their shape. The differences in shrinkage among different colors may be attributed to the pigment types. Although all three pigments are inorganic iron oxide series, their particle sizes and surface characteristics may differ, which could affect packing density and free water content, thereby leading to variations in shrinkage. The average extrusion widths ranged from 6.38 mm to 6.54 mm, with a range of 0.16 mm and a relative standard deviation of 1.1%, which exhibited a stable extrusion width. The average extrusion heights ranged from 5.83 mm to 5.88 mm, with a range of 0.05 mm and a relative standard deviation of 0.4%, which confirmed a stable extrusion height. The extremely small differences in extrusion width (range: 0.16 mm) and extrusion height (range: 0.05 mm) further indicate that the effect of different pigment types on extrusion dimensions is negligible, and the process stability is good. The average compressive strengths ranged from 19.13 N to 20.03 N. The values were tightly clustered, with a low coefficient of variation, indicating that the cured material maintained good stability in compressive strength under the experimental conditions.

In summary, under the current processing parameters, the material achieved stable extrusion. It retained its shape and basic mechanical properties after curing. These results indicate that the material shows potential for fabricating three-dimensional solid models.

### 3.4. Long-Term Performance Evaluation of Extruded Printed Specimens

In this study, the slag–desulfurized gypsum composite was used to print simple multi-layer circular ring stacking specimens. Three colors (red, yellow, and blue, all with a pigment dosage of 6 g) were printed, with three specimens per color, to evaluate the long-term color stability and compressive strength of the material under natural air curing conditions. The printing parameters were consistent with those used in the aforementioned linear printing tests. The printing path was designed as concentric circular rings with a diameter of 80 mm, each layer being an independent closed ring, stacked layer by layer to a total of 13 layers, with a total height of approximately 63 mm. A stacking interval of approximately 30 s was maintained between layers to prevent collapse.

#### 3.4.1. 28-Day Color Stability Test

**Figure 7:**
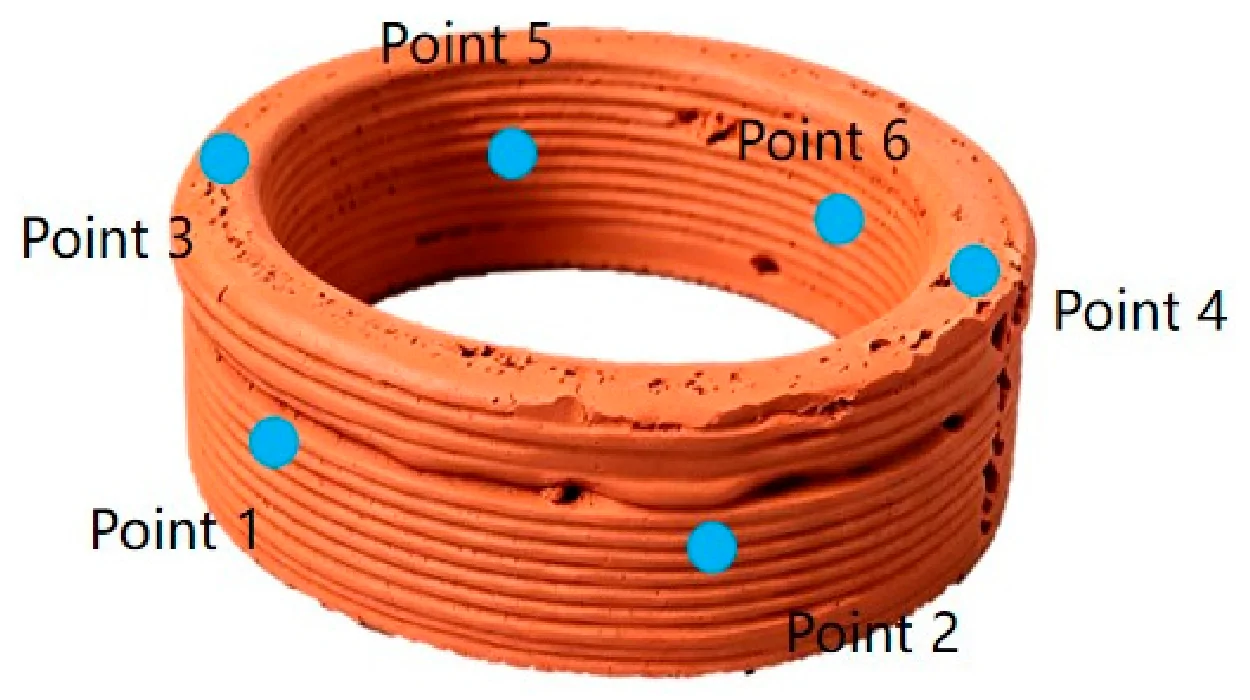
Measurement point locations on the specimens.

Six measurement points were selected on the surface of each specimen (as shown in Figure 7), including two points on the top surface, two points on the outer annular surface, and two points on the inner annular surface (as shown in Figure 7). The colorimetric values at each point were measured immediately after printing completion and after 28 days of natural air curing. The color difference ΔE at each point was calculated according to the CIELAB color difference formula (2) to evaluate the color fading at different positions. The results are presented in Table 12.

After 28 days of natural air curing, the color difference ΔE at various measurement points on each colored specimen remained below 3.0. Specifically, the ΔE ranges for the six measurement points were 1.11–2.59 for the red specimens, 1.22–2.91 for the yellow specimens, and 1.04–2.63 for the blue specimens, indicating that no significant fading occurred in any of the three colored specimens. This can be attributed to the fact that iron oxide pigments are inorganic pigments whose crystal structures exhibit high chemical stability under natural light and room temperature conditions, and are not prone to fading caused by changes in molecular structure. From the perspective of different positions, Points 1 and 2 (sun-exposed surface) exhibited slightly higher ΔE values than Points 5 and 6 (inner annular surface), indicating that the color change on the directly light-exposed surface was slightly greater than that on the relatively shaded surface. This may be related to the fact that the sun-exposed surface receives more ultraviolet radiation from sunlight; however, its promoting effect on color change is limited. Points 3 and 4 (top surface) showed ΔE values between those of the sun-exposed surface and the inner annular surface, further indicating that light intensity is the primary external factor affecting color stability—the stronger the light, the relatively greater the color change. The range of ΔE across the six measurement points for each colored specimen was less than 1.6, indicating that the degree of fading at different positions on the same specimen was relatively uniform. This reflects that the pigment was uniformly dispersed during slurry mixing and printing, which is consistent with the conclusions on pigment dispersion uniformity drawn from the previous color characterization. Based on the above results, the material maintains good color stability under 28 days of natural air curing conditions, preliminarily demonstrating potential for outdoor applications.

#### 3.4.2. 28-Day Compressive Strength Test

The compressive test samples were ring-shaped 3D-printed specimens prepared from blast furnace slag, desulfurized gypsum, and iron oxide pigments. After 28 days of natural air curing, compressive tests were conducted on each of the three specimens per group, and the results were averaged to evaluate the load-bearing performance of the material under 28-day curing conditions. Mechanical property tests were performed using a CMT-1000 electronic universal material testing machine (Fashun, Shaoxing, China) with a maximum capacity of 1 kN and a sensor accuracy of ±0.5%. The loading rate was set at 1 mm/min, and loading was continued until specimen failure. The peak load was recorded as the failure load (N), along with the average value and standard deviation.

According to the test results in Table 13, the average peak loads of the specimens were 24.58 ± 0.54 N for the red group, 23.95 ± 0.51 N for the yellow group, and 23.30 ± 0.49 N for the blue group, with a maximum difference of only 1.28 N among the three groups. This indicates that pigment type has a relatively minor effect on the compressive load of the material, primarily because iron oxide pigments mainly serve a coloring function in the matrix and do not participate in hydration reactions, thus having limited interference with mechanical properties. In addition, the standard deviation ranges for the three specimens within each color group were 0.49–0.54 N, showing a low degree of dispersion, which reflects good process stability during material preparation and printing.

### 3.5. Cost Comparison

Raw material cost is one of the important factors determining the feasibility of promoting and applying new printing materials. To further evaluate the economic potential of the colored slag–desulfurized gypsum composite developed in this study, Table 14 compares the differences among this material, conventional cement-based materials, and commercially available colored cement in terms of raw material cost, energy consumption requirements, and solid waste utilization rate.

In terms of raw material costs, the blast furnace slag and desulfurized gypsum used in this study are both industrial by-products, and their prices are significantly lower than those of ordinary Portland cement in conventional cement-based materials. According to slag quotations from major domestic steel plants, the price of blast furnace slag ranges from approximately 38 to 100 RMB/ton [45]; the transaction price of desulfurized gypsum in the latest Chinese government procurement bidding in May 2026 was approximately 39 RMB/ton [46]; the latest price of iron oxide red pigment in May 2026 was approximately 6500 RMB/ton [47]; and the average price of cement in the Aksu region as of June 2026 was approximately 445 RMB/ton [48]. Based on the mix proportion of this study (62.5% slag, 37.5% gypsum, and 6 g pigment per 100 g of solid raw materials), the comprehensive material cost including pigment was calculated to be approximately 467 RMB/ton. For comparison, the price of commercially available colored cement, such as the 425# colored cement produced by Suzhou Guanghua Cement Plant, ranges from approximately 850 to 1600 RMB/ton [49].

As shown in Table 14, the colored slag–desulfurized gypsum composite developed in this study exhibits a significant advantage in raw material cost. Even after accounting for the cost of iron oxide pigments, its comprehensive cost of approximately 467 RMB/ton remains close to that of conventional cement (approximately 445 RMB/ton), and is far below the lower price limit of commercially available colored cement (approximately 850 RMB/ton). Furthermore, the pigment dosage can be flexibly adjusted within the range of 2–10 g according to actual coloring requirements, with the corresponding comprehensive material cost ranging from approximately 207 to 727 RMB/ton, further enhancing its economic applicability.

Based on the above analysis, the colored slag–desulfurized gypsum composite proposed in this study demonstrates significant advantages in raw material cost. Its comprehensive cost is not only comparable to that of conventional cement-based materials, but also far lower than that of commercially available colored cement. This cost advantage is mainly attributed to the fact that this study uses two types of industrial solid waste, namely blast furnace slag and desulfurized gypsum, as the sole solid raw materials, which not only substantially reduces raw material acquisition costs but also opens up a new pathway for the high-value-added reuse of industrial by-products. By transforming blast furnace slag and gypsum, which originally have low or even negative value, into functional materials with color expression and printability, this study achieves a transformation from solid waste to high-value products. In addition, the flexible adjustment of pigment dosage further enhances its economic applicability, enabling it to maintain good cost competitiveness under different coloring requirements, thereby providing economic feasibility support for the commercial application of colored 3D printing materials. Therefore, this material shows good potential in both cost control and high-value utilization of solid waste, aligning with the development direction of green, low-carbon, and high-value-added 3D printing materials.

## 4. Conclusions

### 4.1. Color Performance and Stability

This study optimized the particle size distribution of SB-3DPM using the Andreasen model. Based on this, an eco-friendly colored SB-3DPM was developed using three iron oxide pigments. A clear threshold was identified: beyond this proportion, the hue and saturation stabilized, and adding more pigment produced no significant visual difference. This finding provides a practical guideline for pigment dosage optimization—it allows the desired color saturation to be achieved with the minimum amount of pigment, thereby avoiding unnecessary material waste and reducing cost without compromising visual quality. The 28-day natural air curing color stability test further demonstrated that the color difference ΔE at various measurement points on each colored specimen remained below 3.0, indicating that the inorganic crystal structure of iron oxide pigments exhibits high chemical stability under natural light and room temperature conditions, ensuring that the material can maintain its aesthetic appearance over time in typical indoor or shaded outdoor environments. These results reveal a deeper insight: iron oxide pigments are not merely decorative additives, but structurally stable components that can withstand prolonged environmental exposure without significant fading. On a practical level, this stability means that color consistency across different parts and batches of printed components can be reliably guaranteed, and the identified pigment threshold provides a standardized basis for formulation management during color switching in production, helping to reduce rework caused by color deviation.

### 4.2. Setting Time and Temperature Effect

Range analysis results showed that water temperature exerted the most significant influence on setting time (range = 255 s), substantially greater than that of pigment dosage (range = 15 s) and pigment type (range = 5 s). This quantitative comparison reveals a hierarchy of control factors: water temperature is the dominant parameter for regulating setting behavior, while pigment-related factors play a secondary role. During 3D printing, adjusting the nozzle temperature and the heated bed temperature accelerated curing, improved the stability of the extruded material, and prevented collapse or deformation of printed models. A higher pigment dosage was also found to accelerate the curing process. By clarifying this hierarchy, operators can proactively coordinate pigment content and temperature settings to maintain consistent print quality across different color formulations, without relying on repeated trial-and-error to determine the process window. In addition, this study also reveals a noteworthy issue: when printing models with high color saturation, the accompanying shortening of curing time should be factored into the overall process parameter considerations—otherwise, premature solidification could compromise interlayer bonding or affect smooth nozzle discharge.

### 4.3. Rheological Behavior and Printability

The results showed that when the shear rate exceeded 10^−1^ s^−1^, the viscosity of all three colored slurries dropped below 50 Pa·s. In this range, stable and continuous extrusion through the nozzle was achieved. The prepared slurries exhibited clear shear-thinning behavior, which follows the Herschel–Bulkley model, with the flow index *n* between 0 and 1, satisfying the rheological requirements for extrusion-based 3D printing. Extrusion printing tests further confirmed that the strip-shaped specimens of the three colors were extruded continuously and uniformly, without fracture or intermittent extrusion. Quantitatively, the range of extrusion width was only 0.16 mm, and the range of extrusion height was only 0.05 mm, indicating small dimensional fluctuations and demonstrating that the material can produce printed components with high geometric precision and cross-color consistency. It is particularly worth pointing out that the incorporation of iron oxide pigments did not compromise the rheological printability of the SB-3DPM system. The establishment of this premise removes a key obstacle for multi-color 3D printing, meaning that within the same print job, a single set of extrusion parameters can be used across different colors, which simplifies operational procedures and ensures uniform geometric accuracy among different color regions, avoiding misaligned joints or interlayer offsets caused by frequent parameter switching.

### 4.4. Mechanical Properties

The 28-day compressive strength test results showed that the average compressive strengths of the three colored specimen groups ranged from 23.30 to 24.58 N, representing an increase of approximately 20–25% over the early-age strength of single-layer specimens. The effect of pigment type on strength was relatively small (maximum difference of only 1.28 N), and the standard deviation within each color group ranged from 0.49 to 0.54 N, showing a low degree of dispersion, which reflects good stability in both material preparation and printing processes. From these data, an important insight can be drawn: the mechanical performance of the colored material is primarily governed by the base mix design rather than by pigment selection. It is precisely this decoupling between color and strength that allows designers to freely choose colors based on aesthetic or functional requirements without sacrificing load-bearing capacity, which greatly simplifies material formulation design for different application scenarios. At the same time, when printing multi-color composite components, the mechanical properties across different color regions can remain largely consistent, effectively avoiding local weak points caused by strength differences and providing assurance for structural safety and reliability.

### 4.5. Cost and Economic Feasibility

Based on the mix proportion of this study (62.5% slag, 37.5% gypsum, and 6 g pigment per 100 g of solid raw materials), the comprehensive material cost including pigment was calculated to be approximately 467 RMB/ton. This is comparable to that of ordinary cement but far lower than that of commercially available colored cement (850–1600 RMB/ton). The economic benefit arises directly from the resource utilization of blast furnace slag—an industrial solid waste that traditionally carries extremely low or even negative disposal costs. Through this research, the slag has been transformed into a product whose value is comparable to that of traditional cement, representing a significant added-value leap. This transformation of “waste into treasure” is the core embodiment of high-value-added reuse of solid waste. For practical promotion, the significance of this cost advantage is multifaceted: compared with commercially available colored cement, the cost reduction of approximately 45–71% provides ample pricing flexibility for entering price-sensitive landscape decoration and large-scale customization markets. Furthermore, the solid-waste-based formulation reduces dependence on natural resources, which aligns with green building and circular economy trends and can enhance the environmental added value and market acceptance of printed products.

### 4.6. Application Prospects and Future Work

Combined with the demonstrated mechanical properties, color stability, and cost advantages, this material has preliminarily proven feasible for replacing traditional colored cement products in certain non-load-bearing decorative scenarios. Specifically, it can be preferentially applied to non-load-bearing landscape components with decorative color requirements, such as garden ornaments, urban furniture, and architectural decorative panels, and can also serve as a printing material for small-scale structures such as outdoor sculptures and signage. From a broader perspective, the greater contribution of this study lies in establishing a complete pathway from solid waste to high-value decorative 3D printing materials—a model that can be extended to other waste streams and pigment systems. In the future, to further promote the outdoor practical application of this material, the durability and serviceability of structures produced with this material should be systematically investigated, including resistance to cleaning, washing, rain, frost, and sun exposure, so as to comprehensively evaluate their long-term performance in real service environments.

## Figures and Tables

**Table 1:** Comparison of ideal and measured particle size distributions.

No.	Representative Particle Size D (μm)	Ideal Cumulative Mass Ptarget (D) (%)	Measured Cumulative Mass (%)	Standard Deviation (SD, n = 3) of the Measured Values (%)
1	10 μm	44.65%	37.31%	≤1.1%
2	20 μm	60.99%	67.42%	≤0.9%
3	30 μm	73.20%	80.17%	≤0.8%
4	40 μm	83.32%	88.36%	≤1.0%
5	50 μm	92.12%	96.51%	≤0.7%
6	60 μm	100%	100%	≤0.5%

**Table 2:** Average L*, a*, b* values of specimens colored with Pigment Red 101 at different dosages (average ± SD, n = 18).

Pigment Red 101	Specimen 1, 2, and 3			L* (Average ± SD)	a* (Average ± SD)	b* (Average ± SD)
Add 2 g	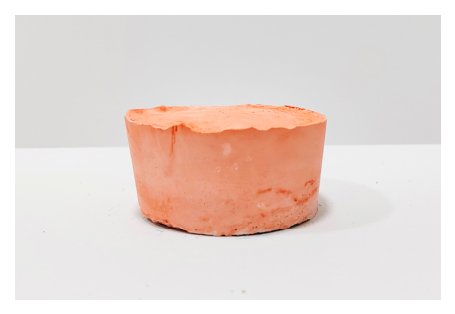	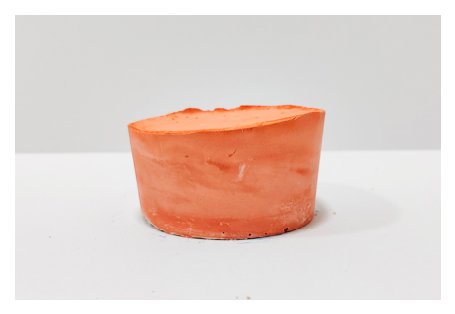	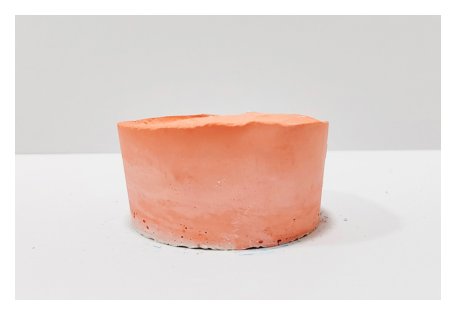	64.22 ± 2.2	24.11 ± 1.8	26.50 ± 1.6
Add 6 g	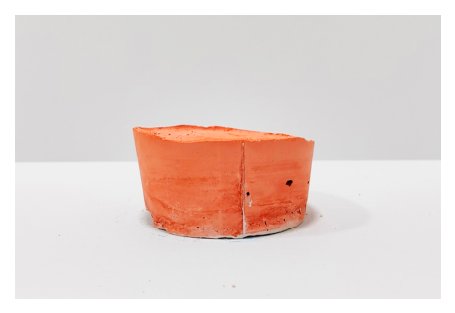	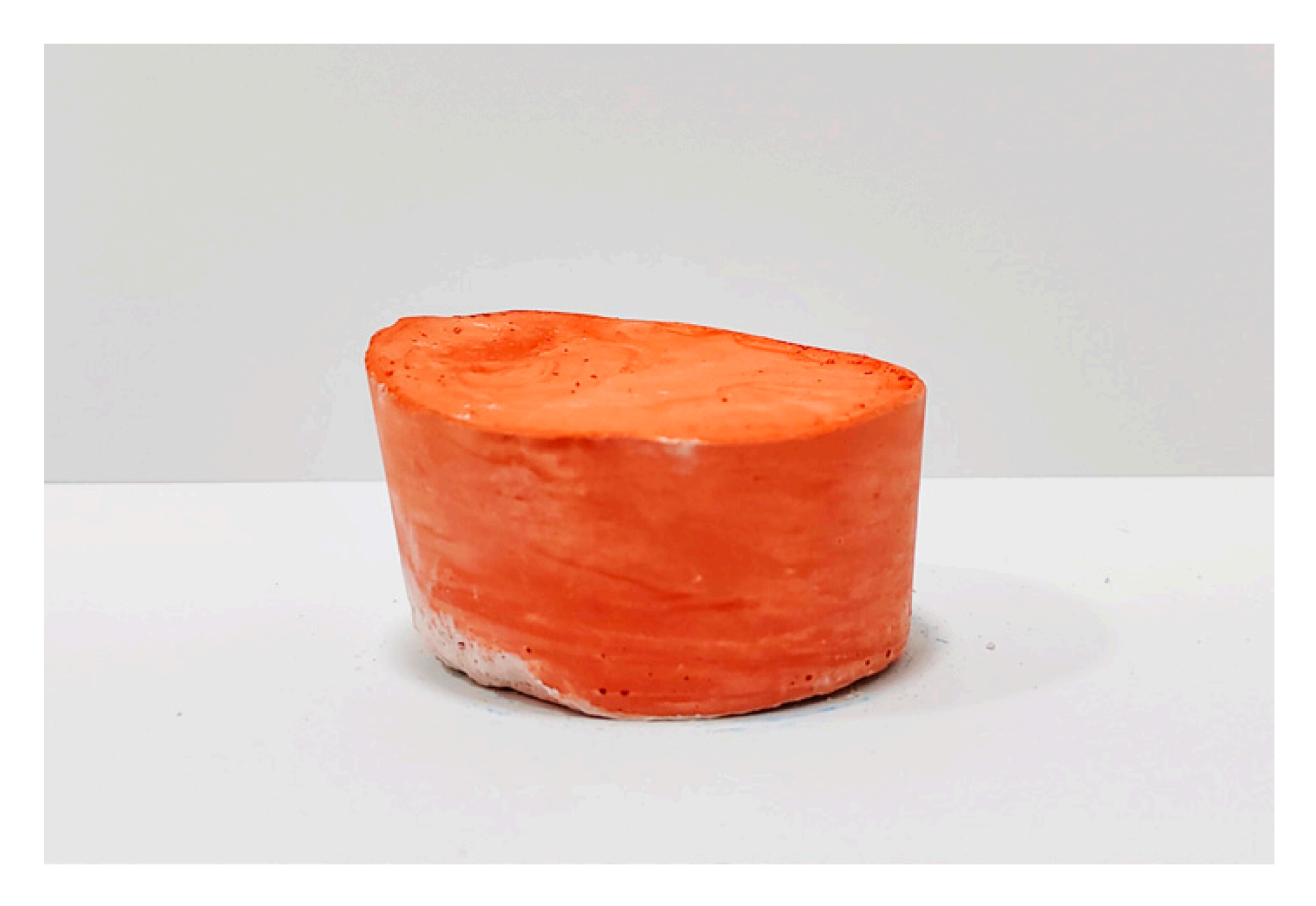	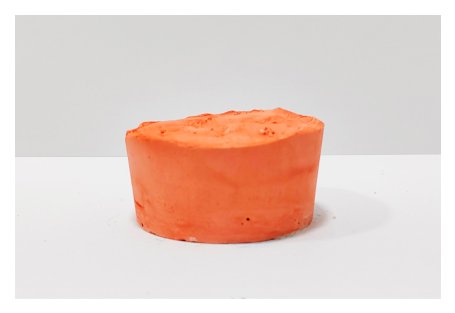	53.11 ± 2.8	40.57 ± 2.0	40.72 ± 1.9
Add 10 g	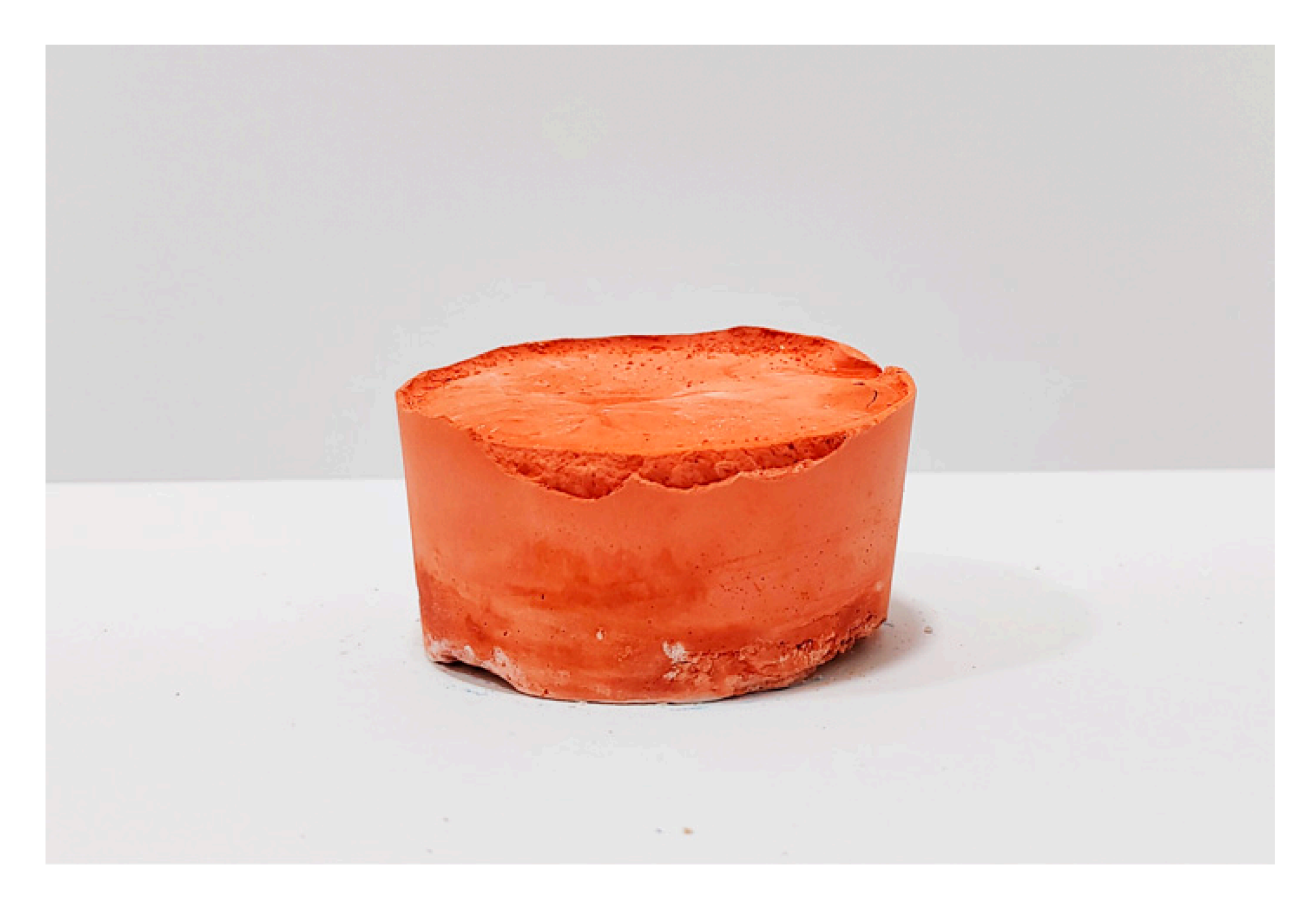	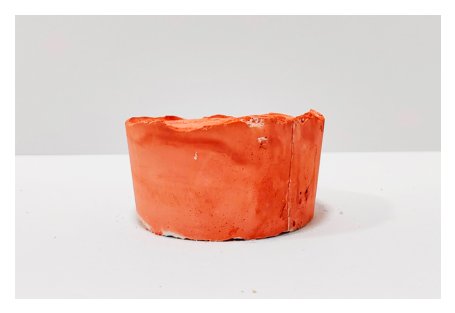	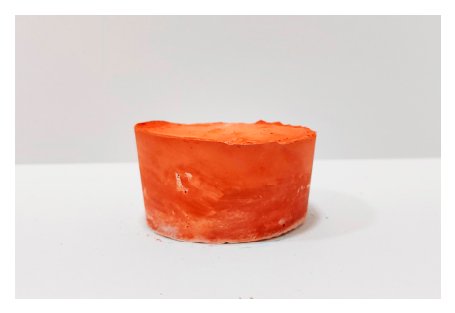	52.57 ± 2.5	43.00 ± 2.2	42.67 ± 2.0

**Table 3:** Average L*, a*, b* values of specimens colored with Pigment Yellow 42 at different dosages (average ± SD, n = 18).

Pigment Yellow 42	Specimen 1, 2, and 3			L* (Average ± SD)	a* (Average ± SD)	b* (Average ± SD)
Add 2 g	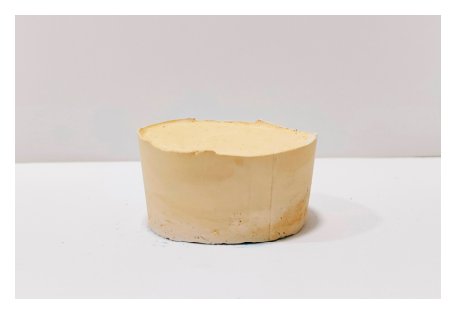	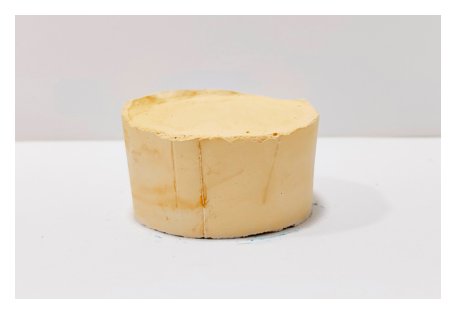	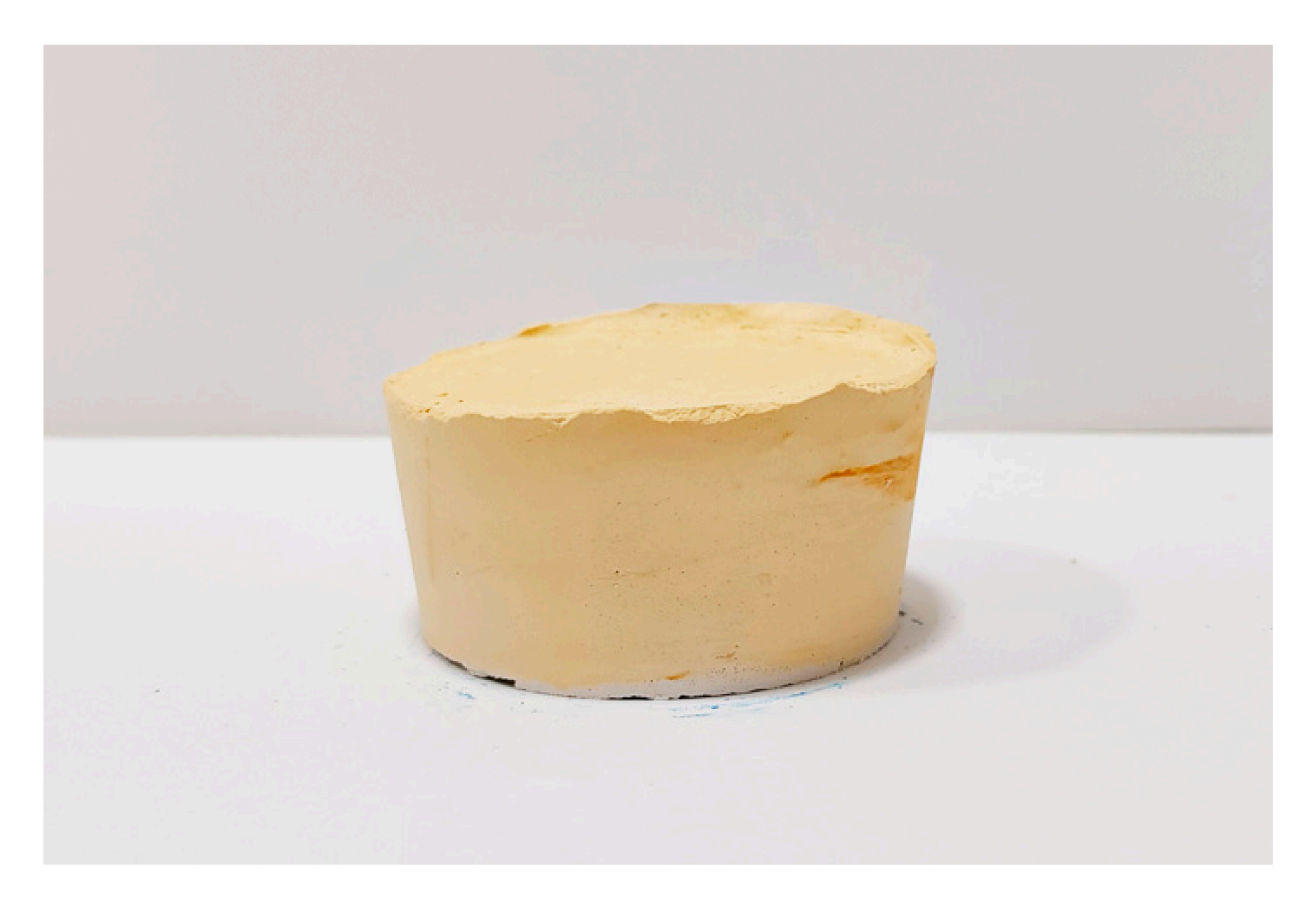	72.61 ± 1.8	4.61 ± 1.6	27.00 ± 1.7
Add 6 g	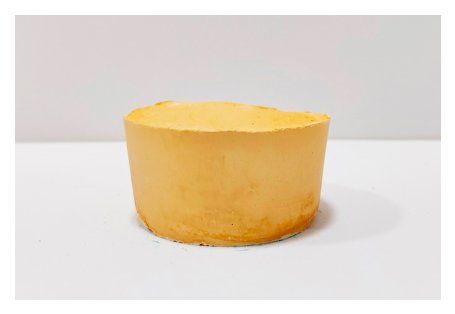	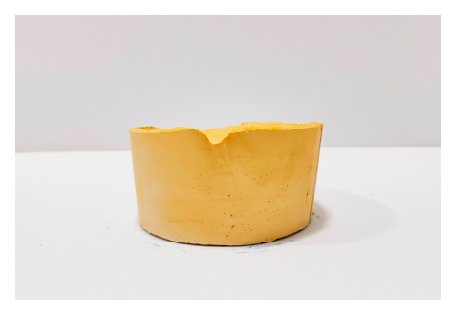	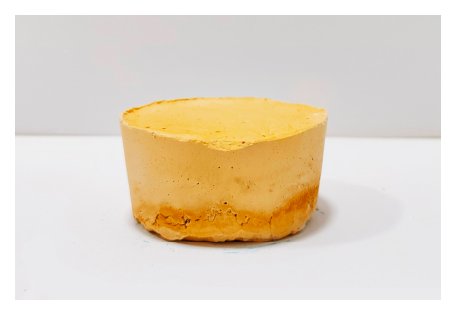	41.34 ± 3.2	8.72 ± 2.1	47.28 ± 2.4
Add 10 g	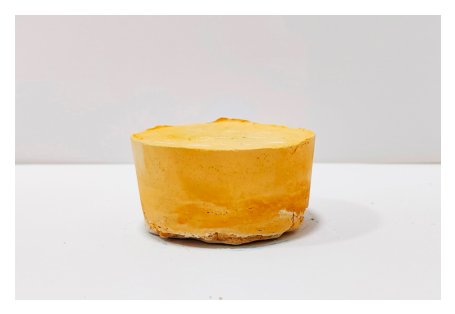	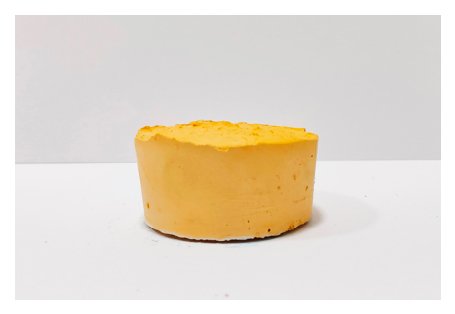	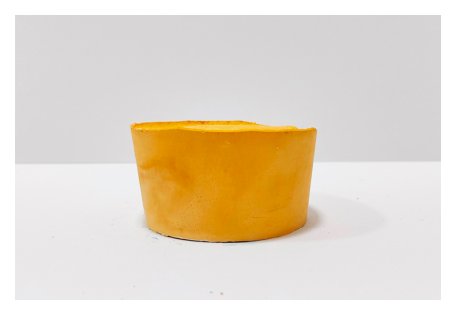	68.83 ± 2.3	13.72 ± 1.9	57.16 ± 2.2

**Table 4:** Average L*, a*, b* values of specimens colored with Pigment Blue 27 at different dosages (average ± SD, n = 18).

Pigment Blue 27	Specimen 1, 2, and 3			L* (Average ± SD)	a* (Average ± SD)	b* (Average ± SD)
Add 2 g	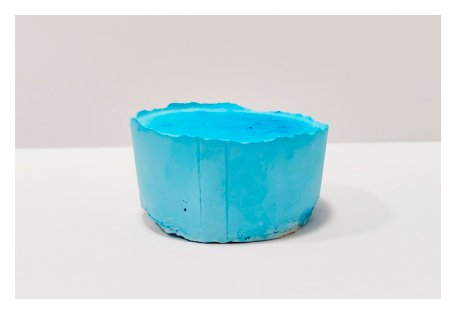	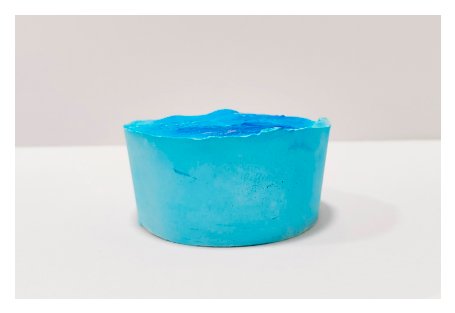	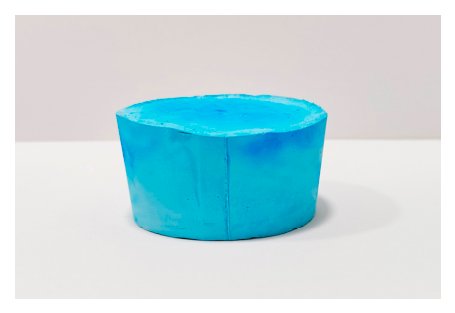	68.55 ± 1.9	−21.11 ± 1.7	−20.83 ± 1.8
Add 6 g	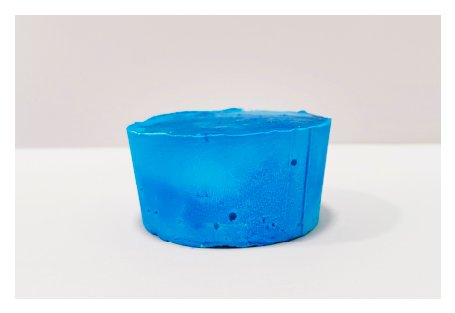	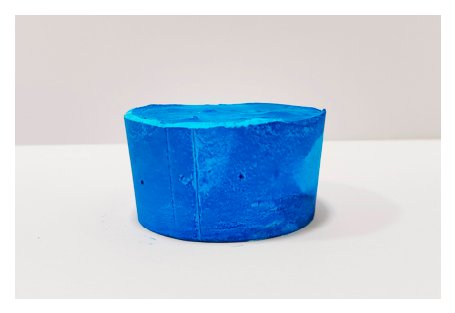	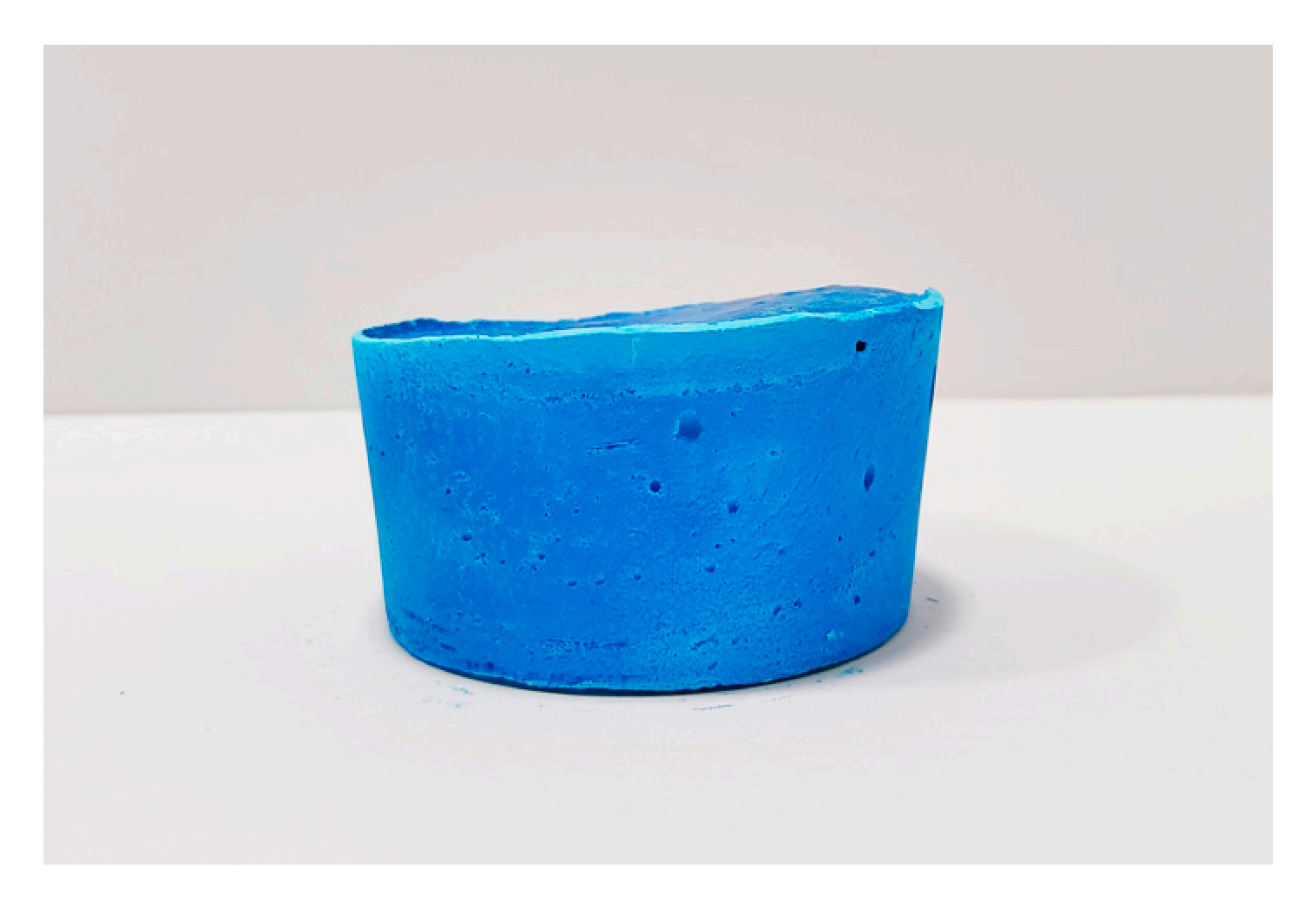	47.66 ± 3.0	−9.50 ± 2.2	57.22 ± 2.6
Add 10 g	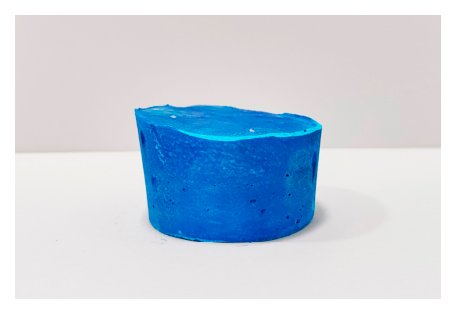	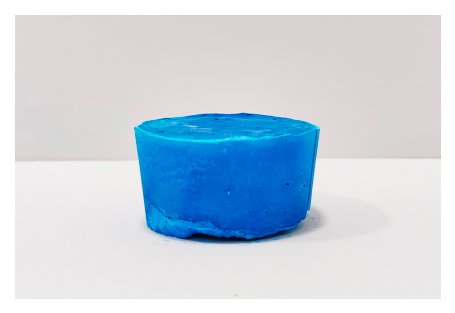	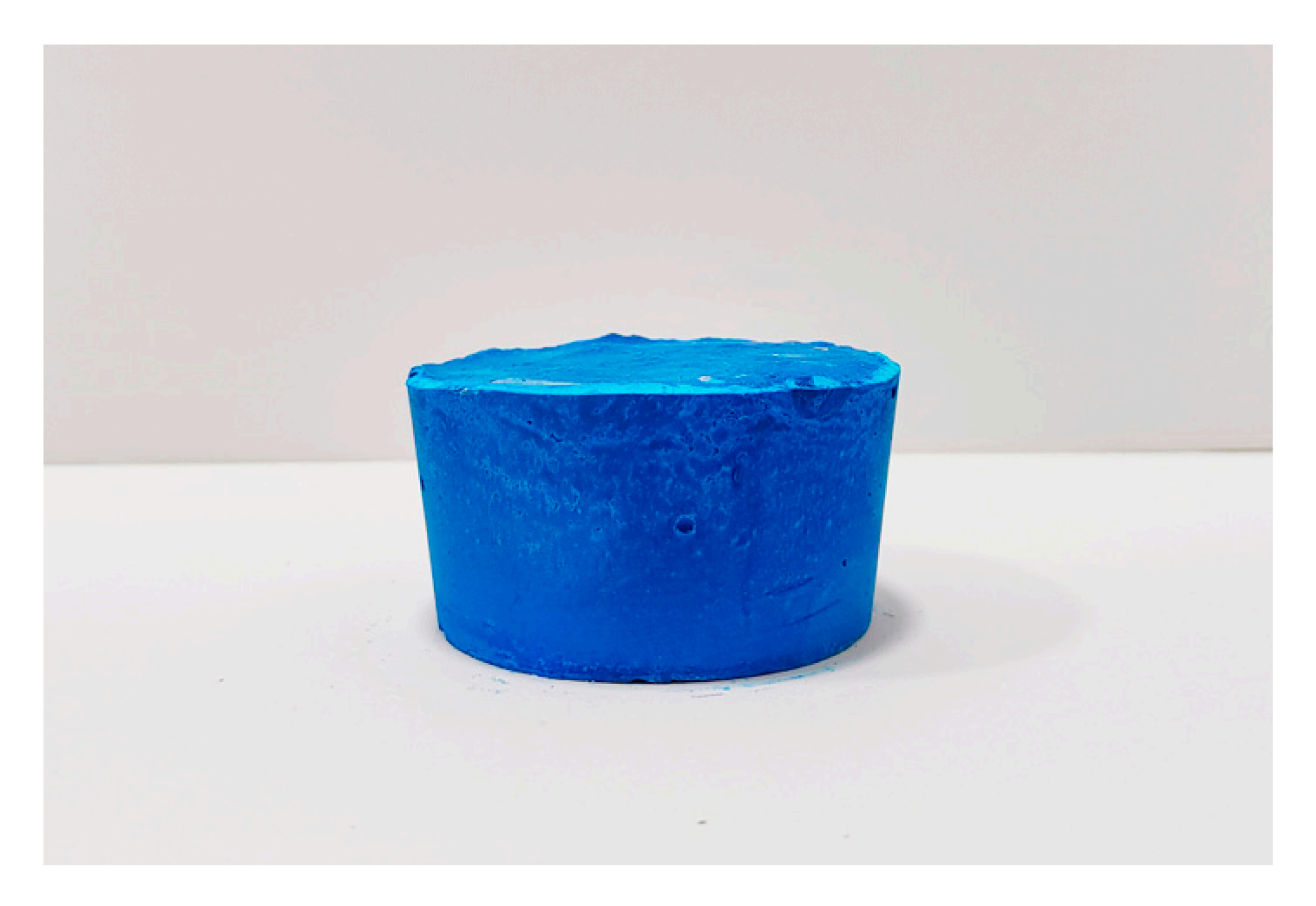	37.72 ± 2.7	6.49 ± 2.0	59.17 ± 2.5

**Table 5:** Color difference ΔE of specimens with different pigment dosages relative to the uncolored reference specimen.

Specimen	ΔE with 2 g Added	ΔE with 6 g Added	ΔE with 10 g Added
Specimen colored with Pigment Red 101	34.82	59.14	62.2
Specimen colored with Pigment Yellow 42	24.45	57.35	55.93
Specimen colored with Pigment Blue 27	34.35	62.63	68.75

**Table 6:** Three-channel statistics for the red specimens.

	Captured Image of the Red Specimen	Red Channel Image	Green Channel Image	Blue Channel Image
Captured image 1	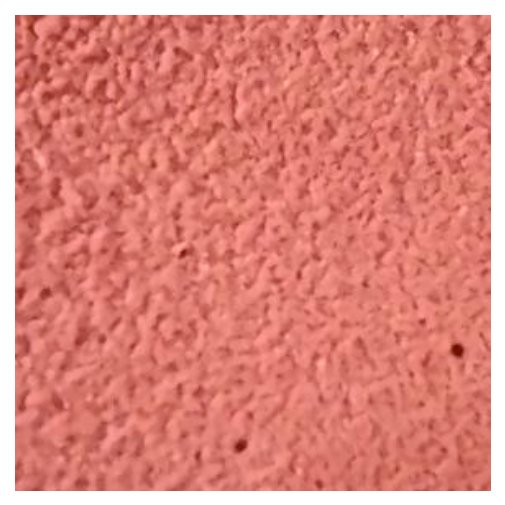	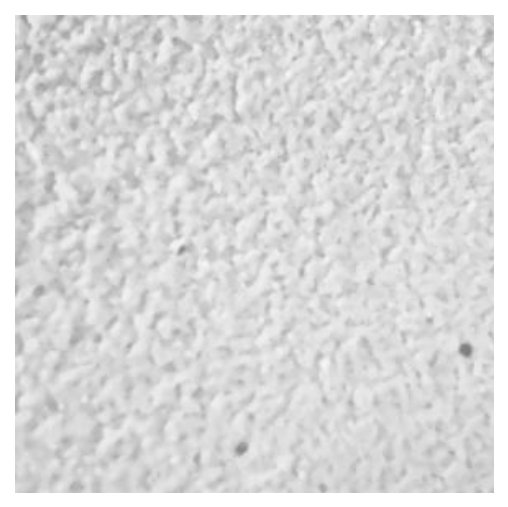	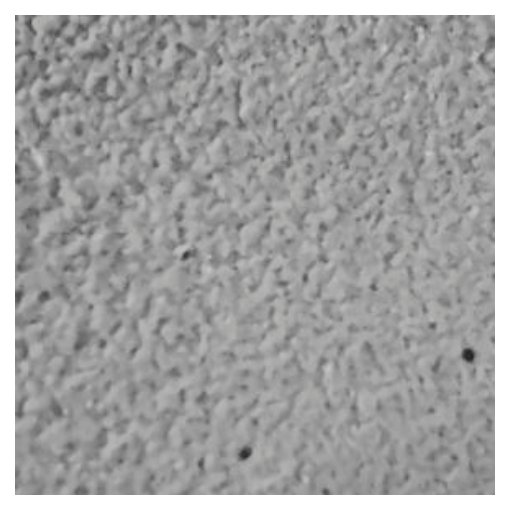	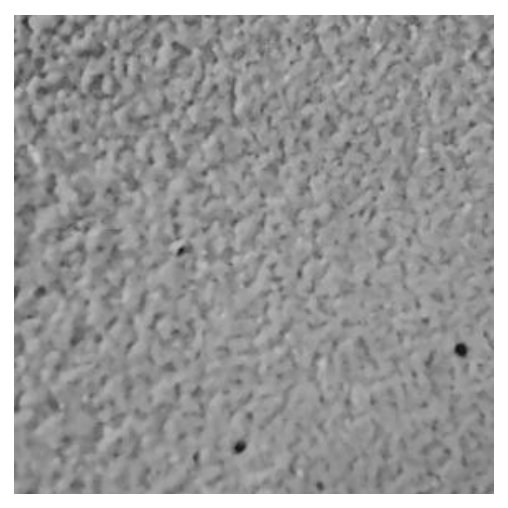
Intensity	138.95	212.57	150.05	141.31
Captured image 2	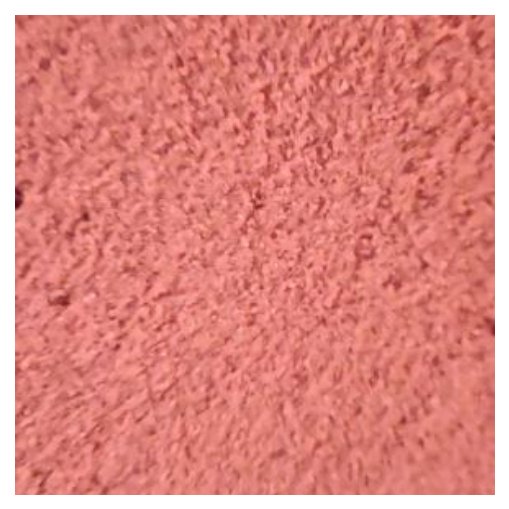	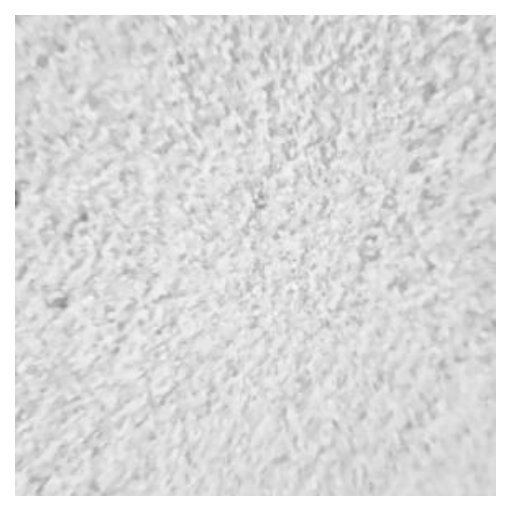	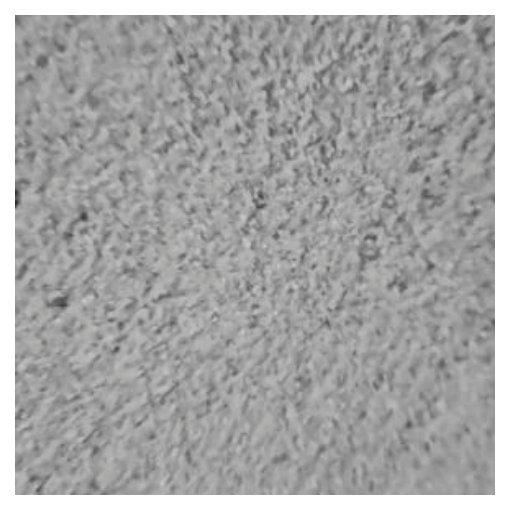	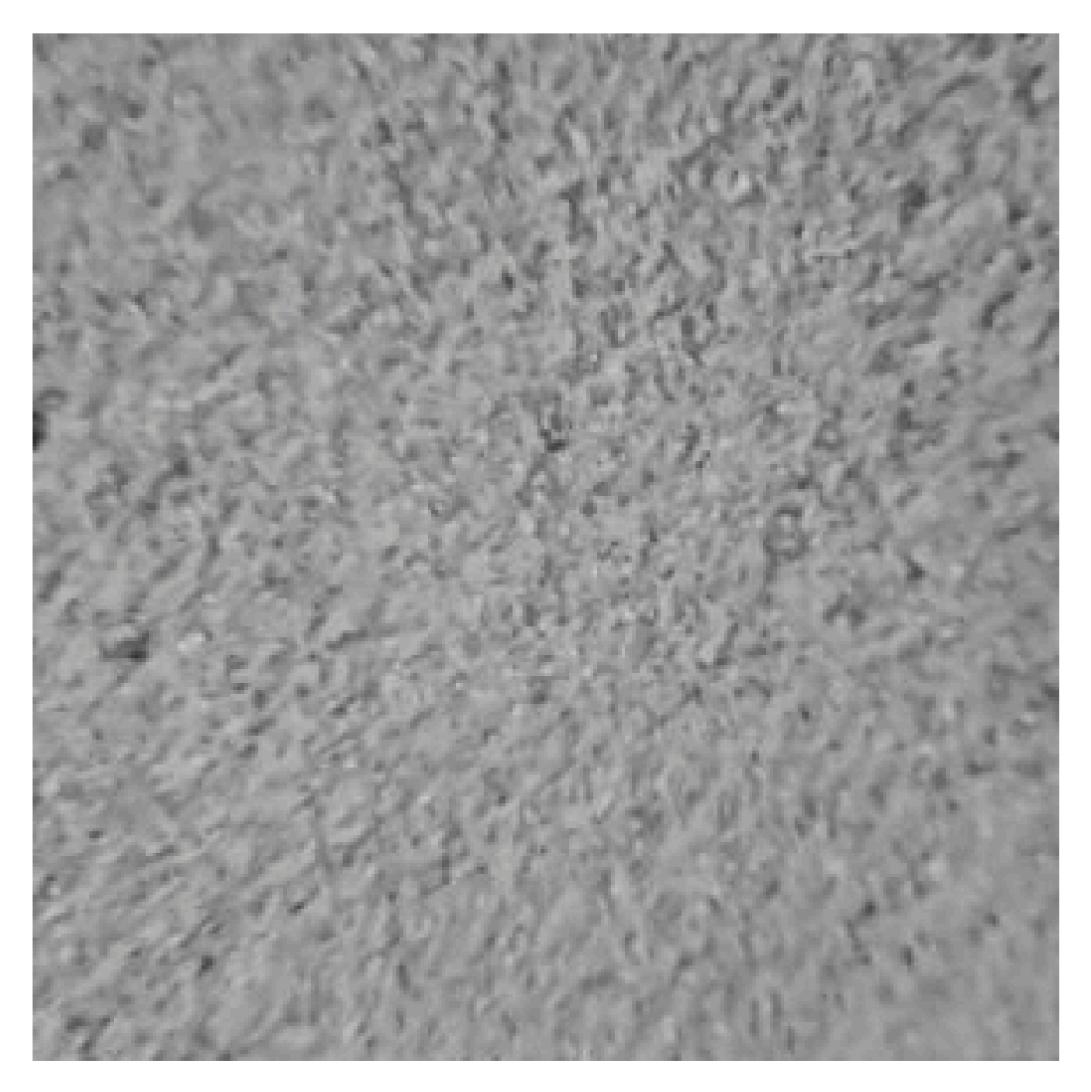
Intensity	142.81	214.22	153.74	147.60
Captured image 3	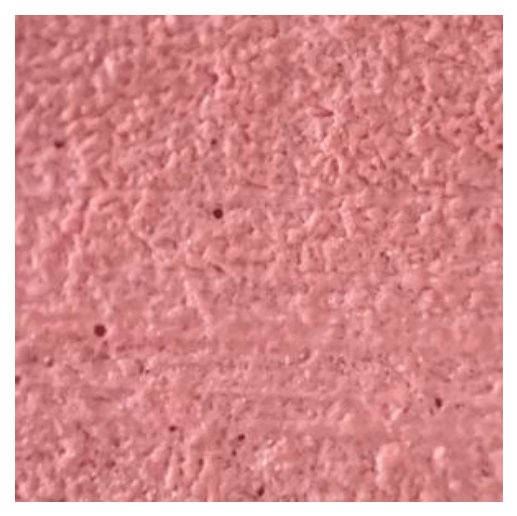	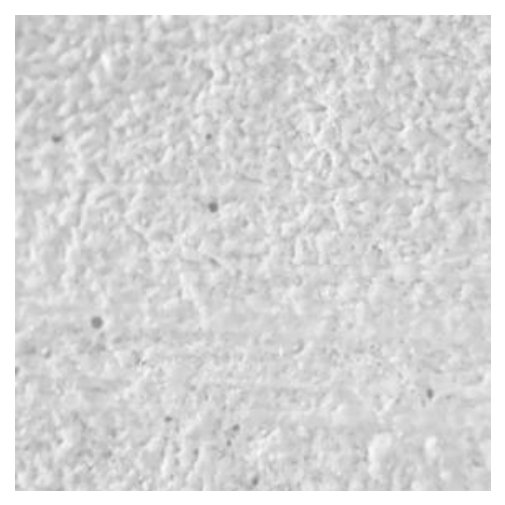	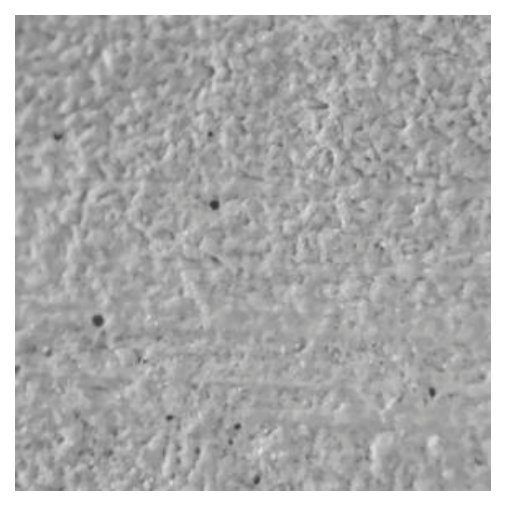	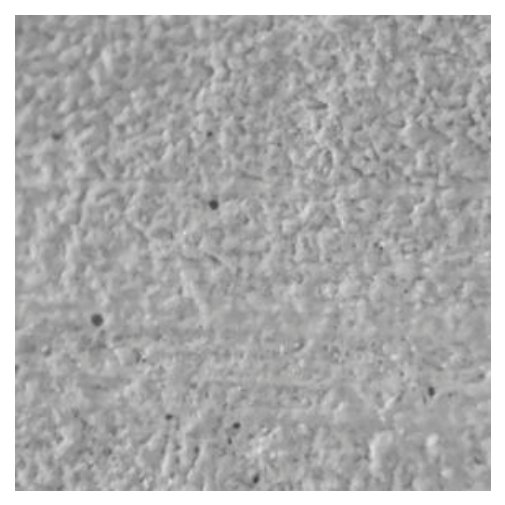
Intensity	147.37	209.64	159.99	161.08

**Table 7:** Three-channel statistics for the yellow specimen.

	Captured Image of the Yellow Specimen	Red Channel Image	Green Channel Image	Blue Channel Image
Captured image 1	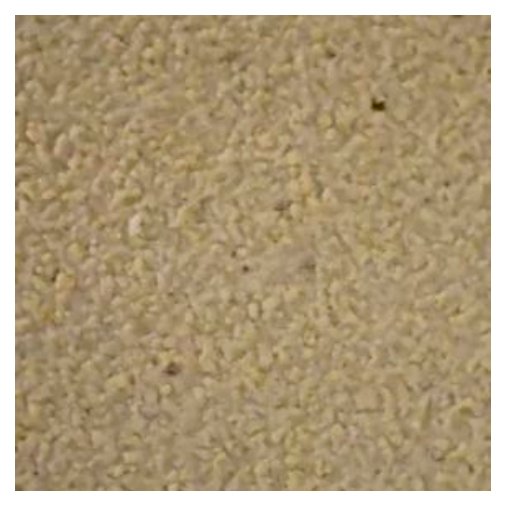	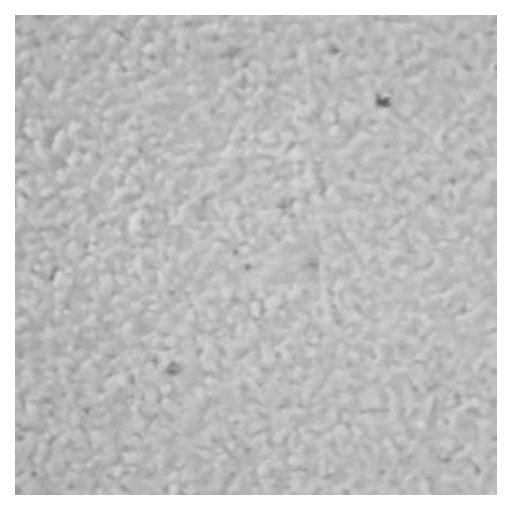	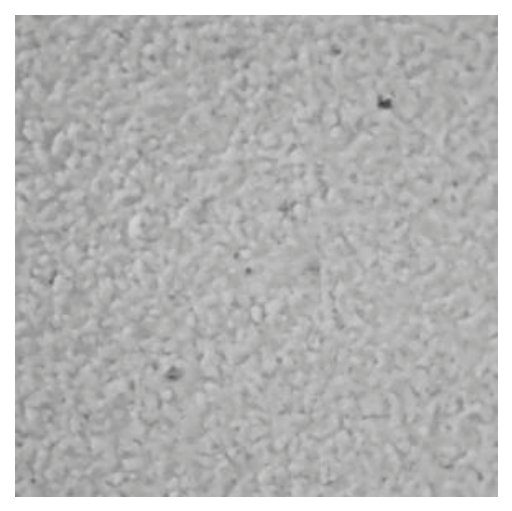	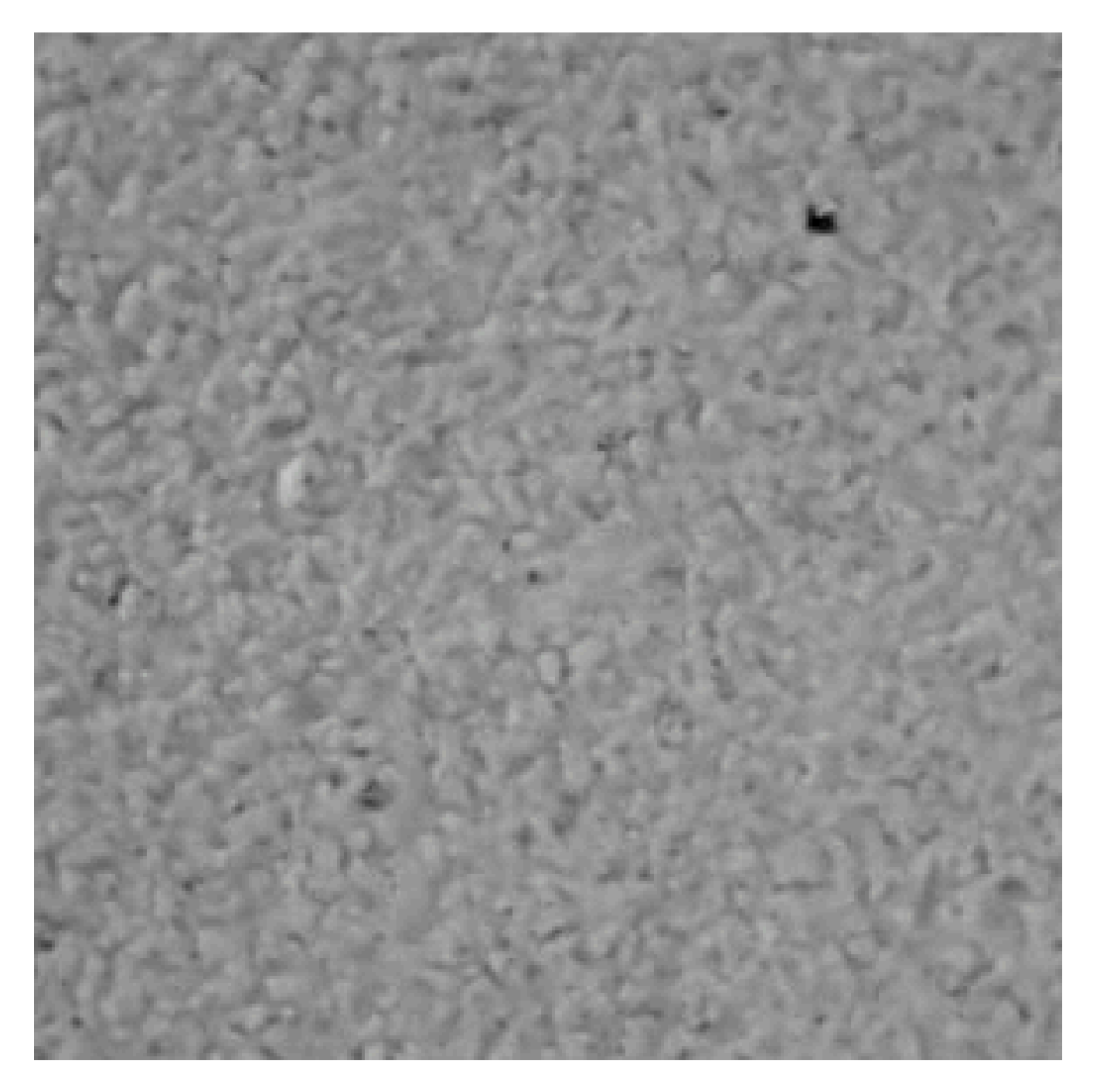
Intensity	142.92	183.98	171.91	142.01
Captured image 2	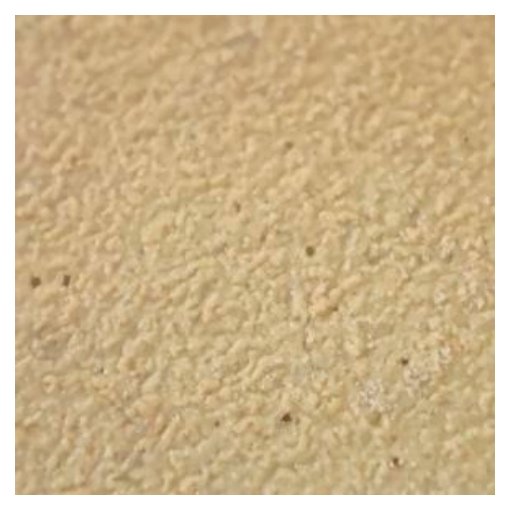	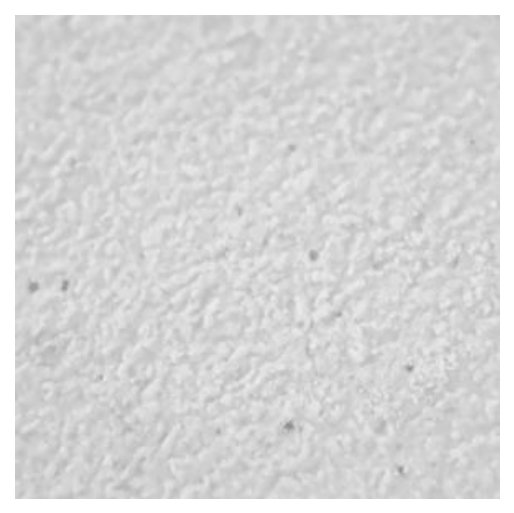	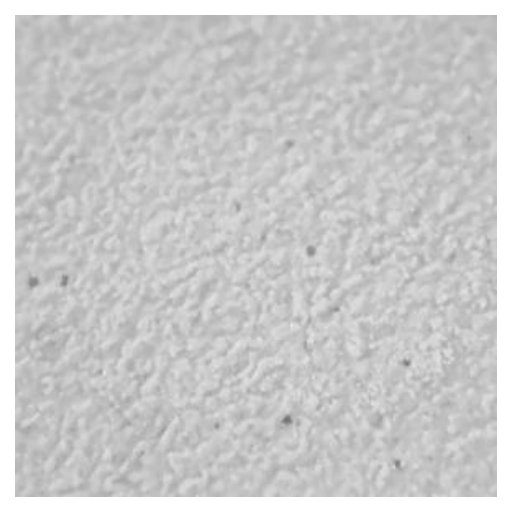	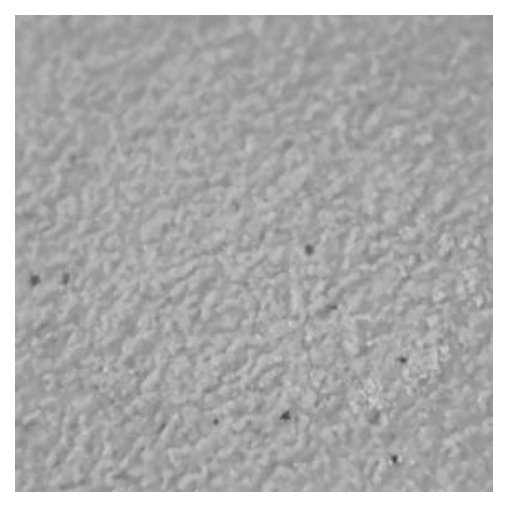
Intensity	173.27	210.38	195.82	162.50
Captured image 3	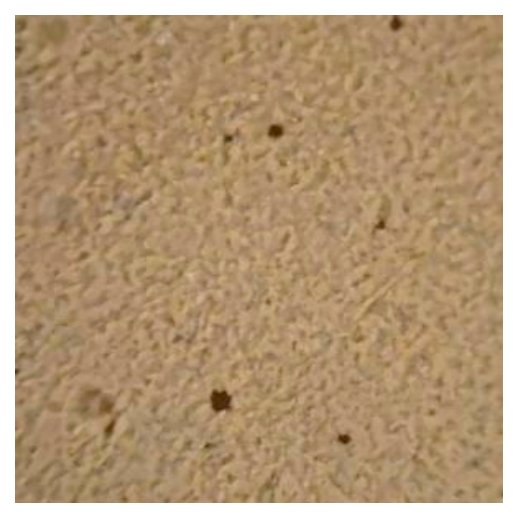	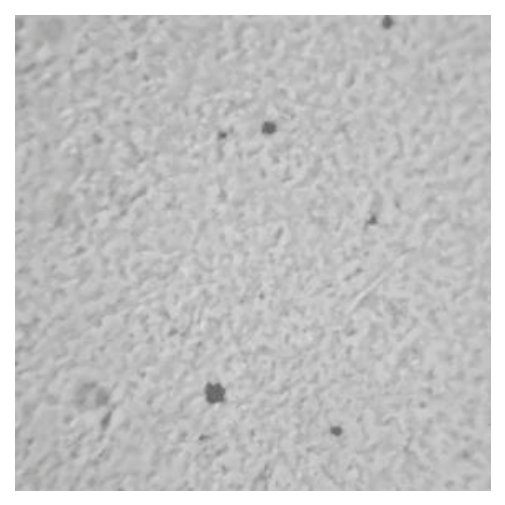	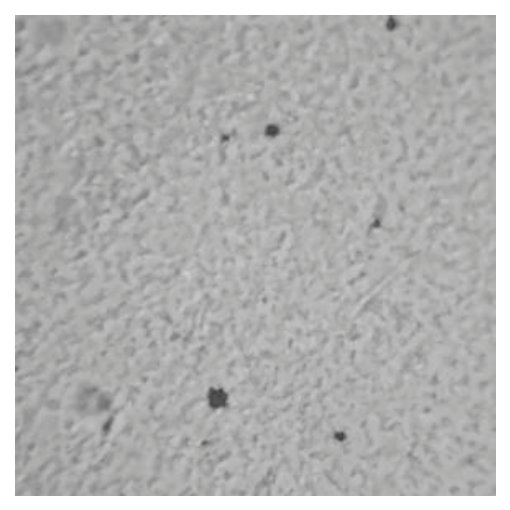	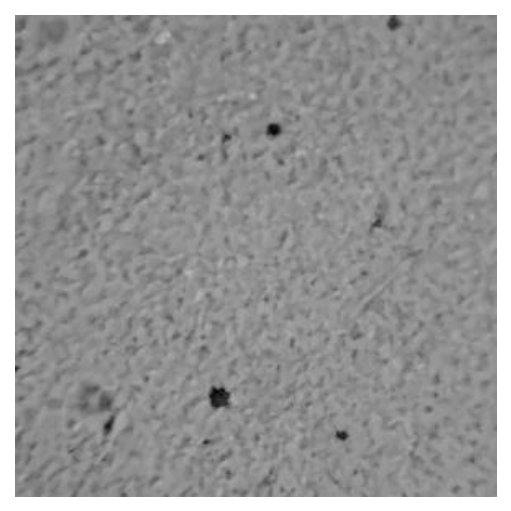
Intensity	141.61	186.69	169.45	138.59

**Table 8:** Three-channel statistics for the blue specimen.

	Captured Image of the Blue Specimen	Red Channel Image	Green Channel Image	Blue Channel Image
Captured image 1	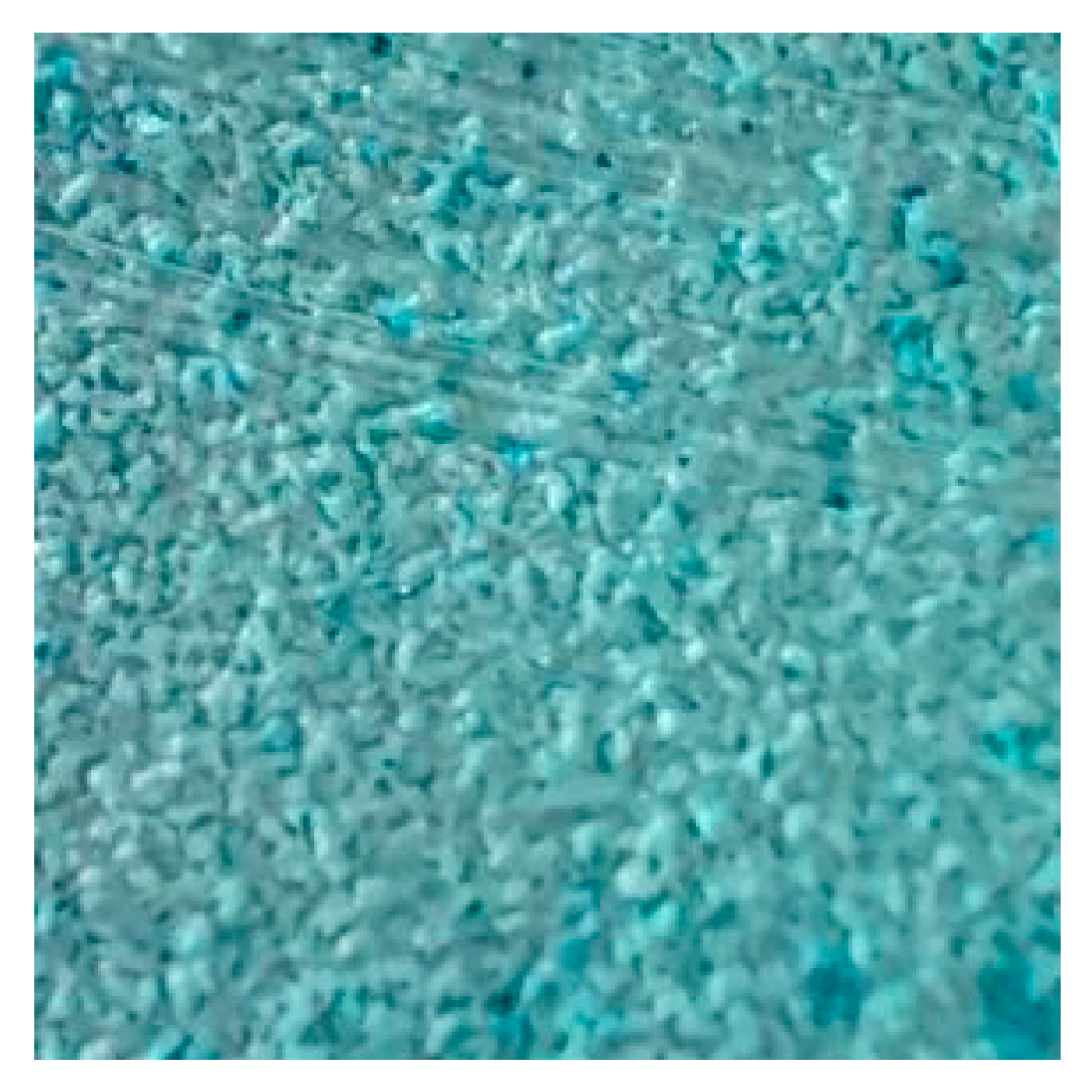	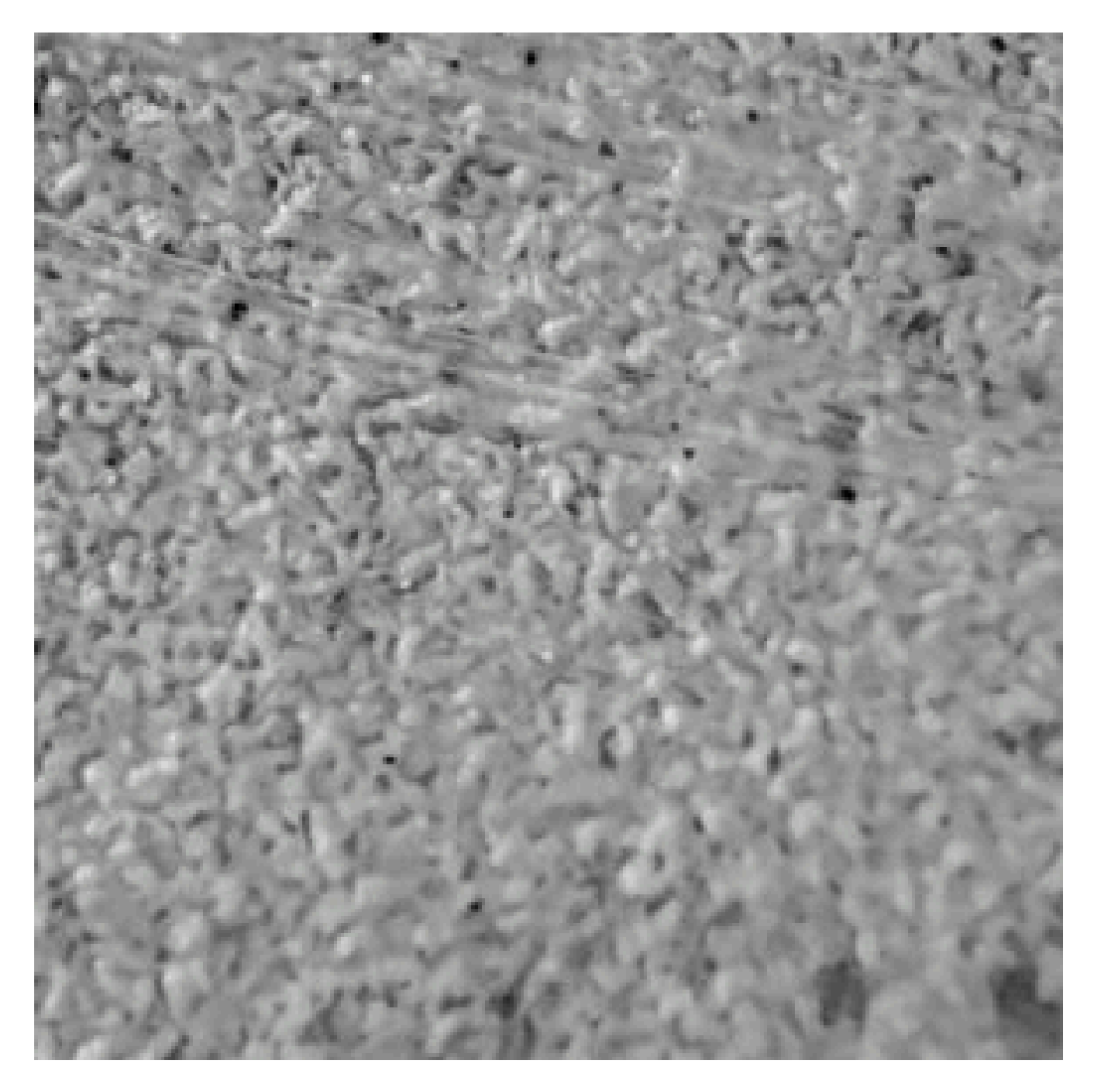	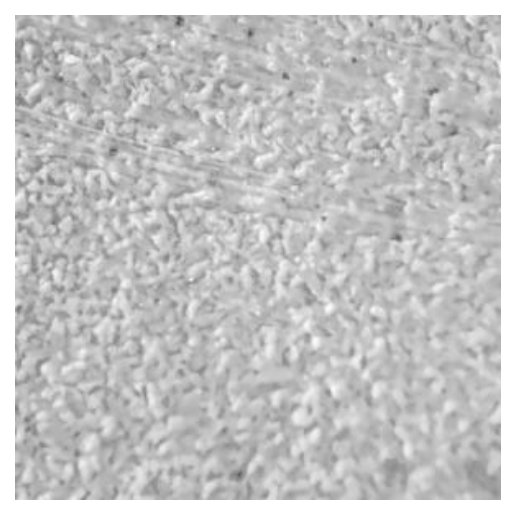	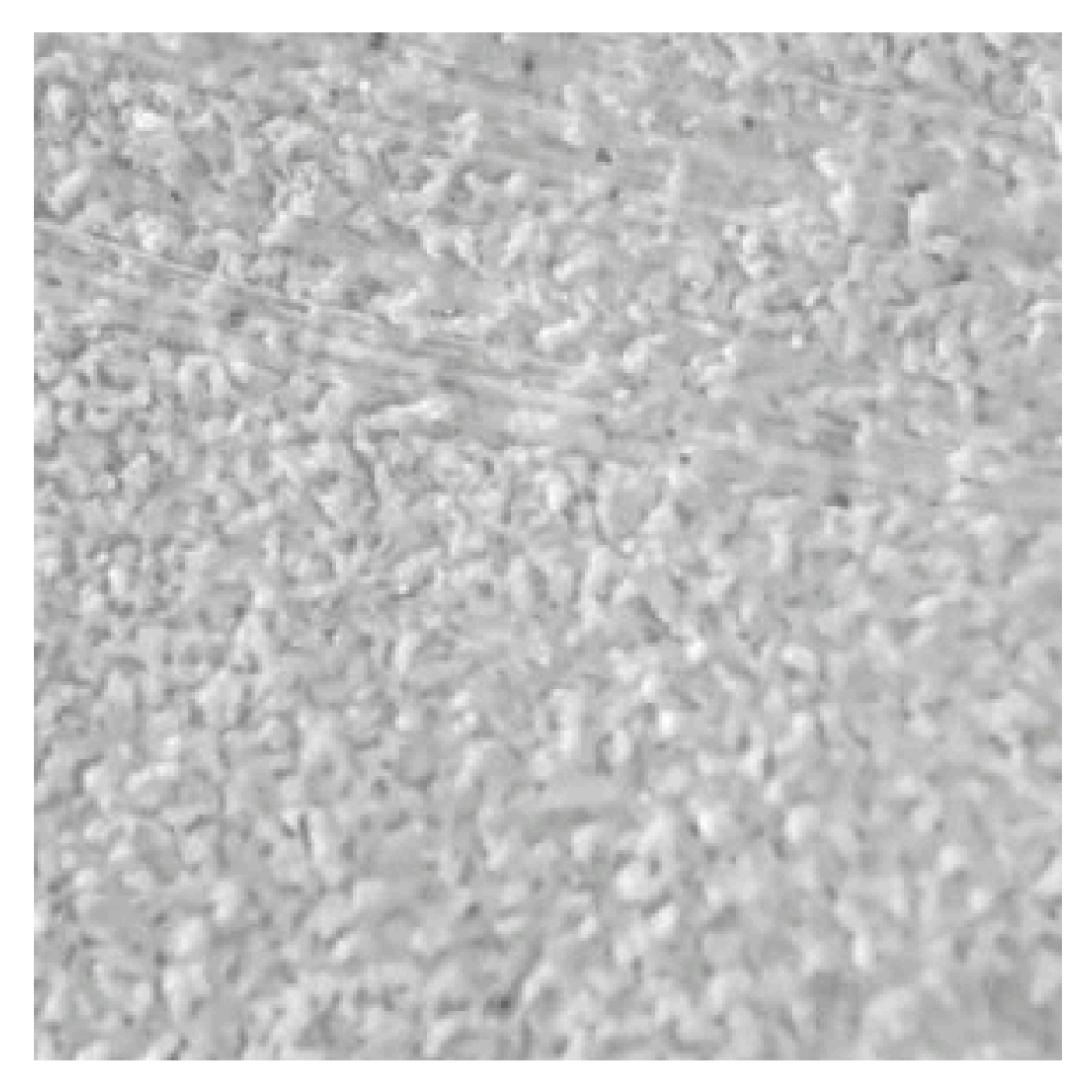
Intensity	148.54	147.09	188.20	189.88
Captured image 2	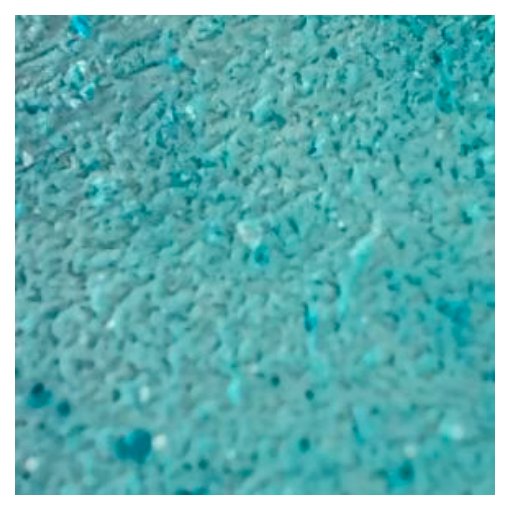	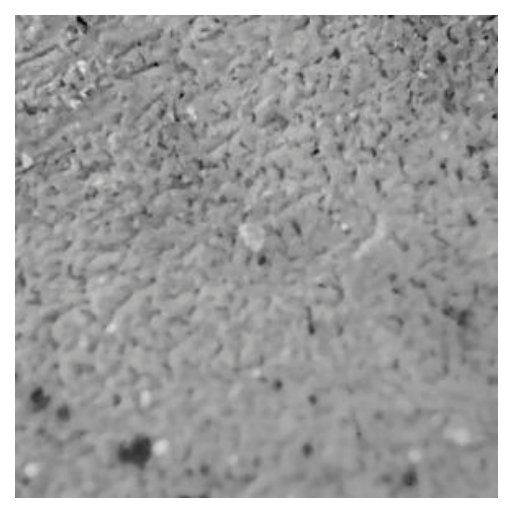	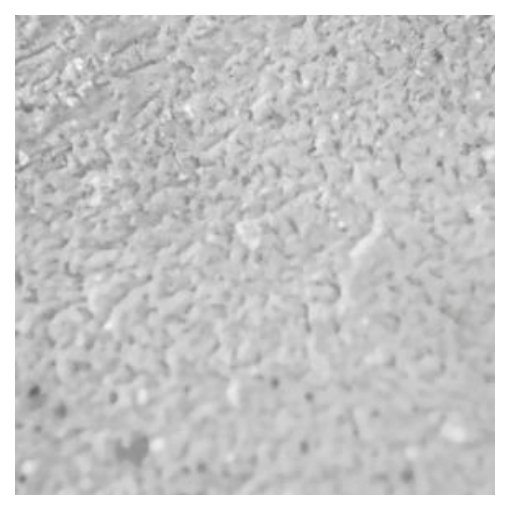	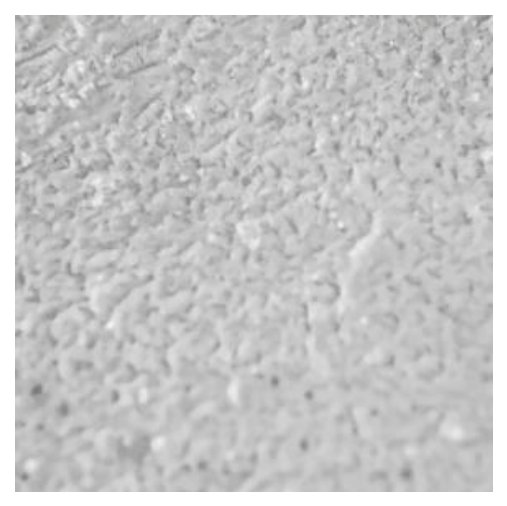
Intensity	150.35	146.33	190.99	192.09
Captured image 3	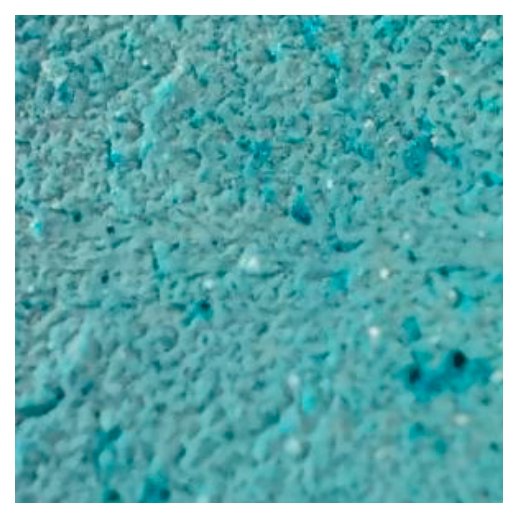	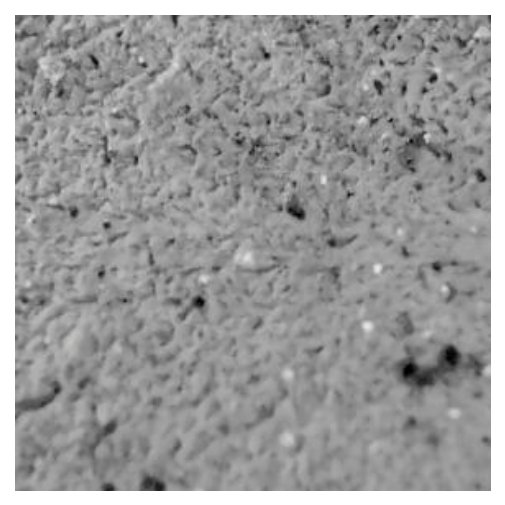	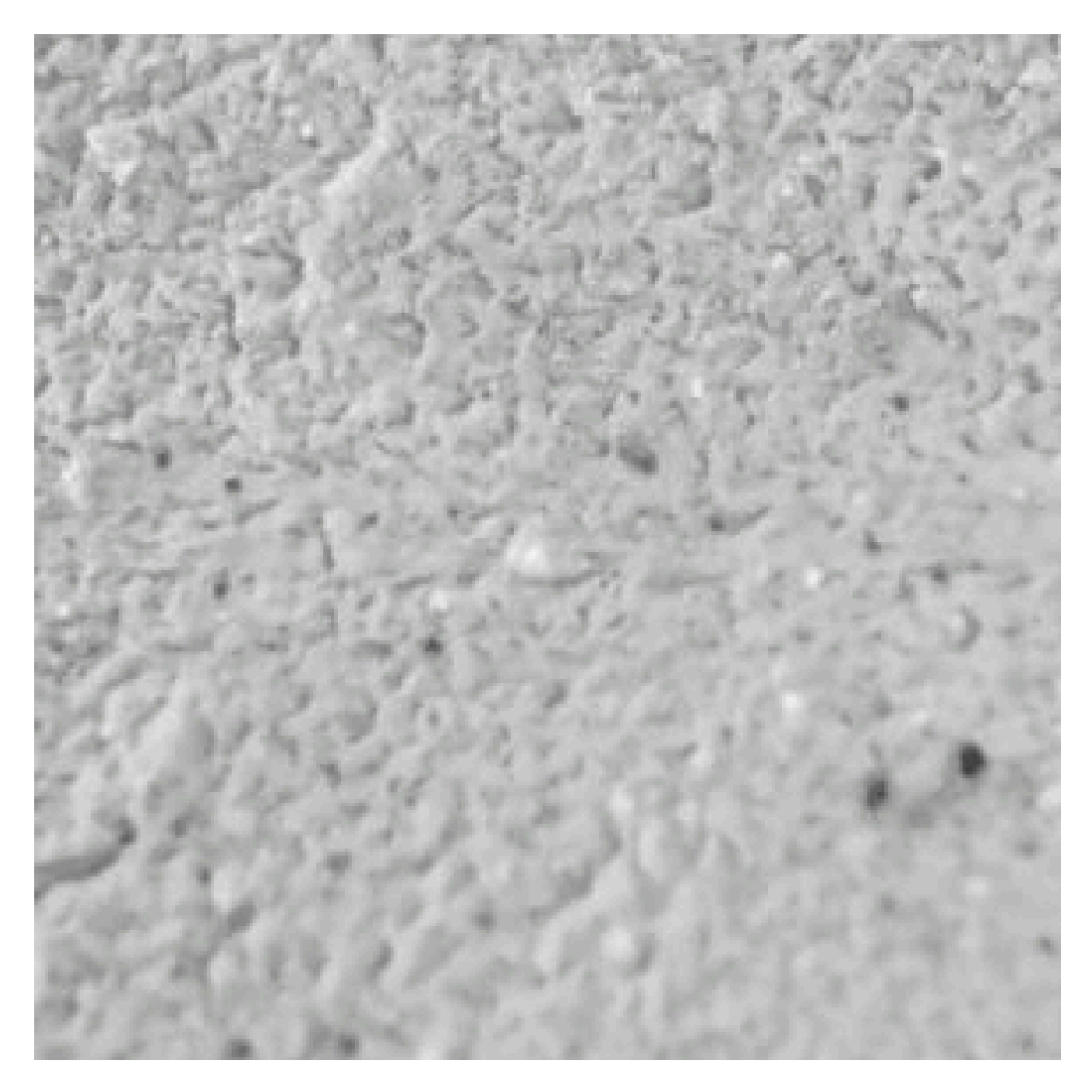	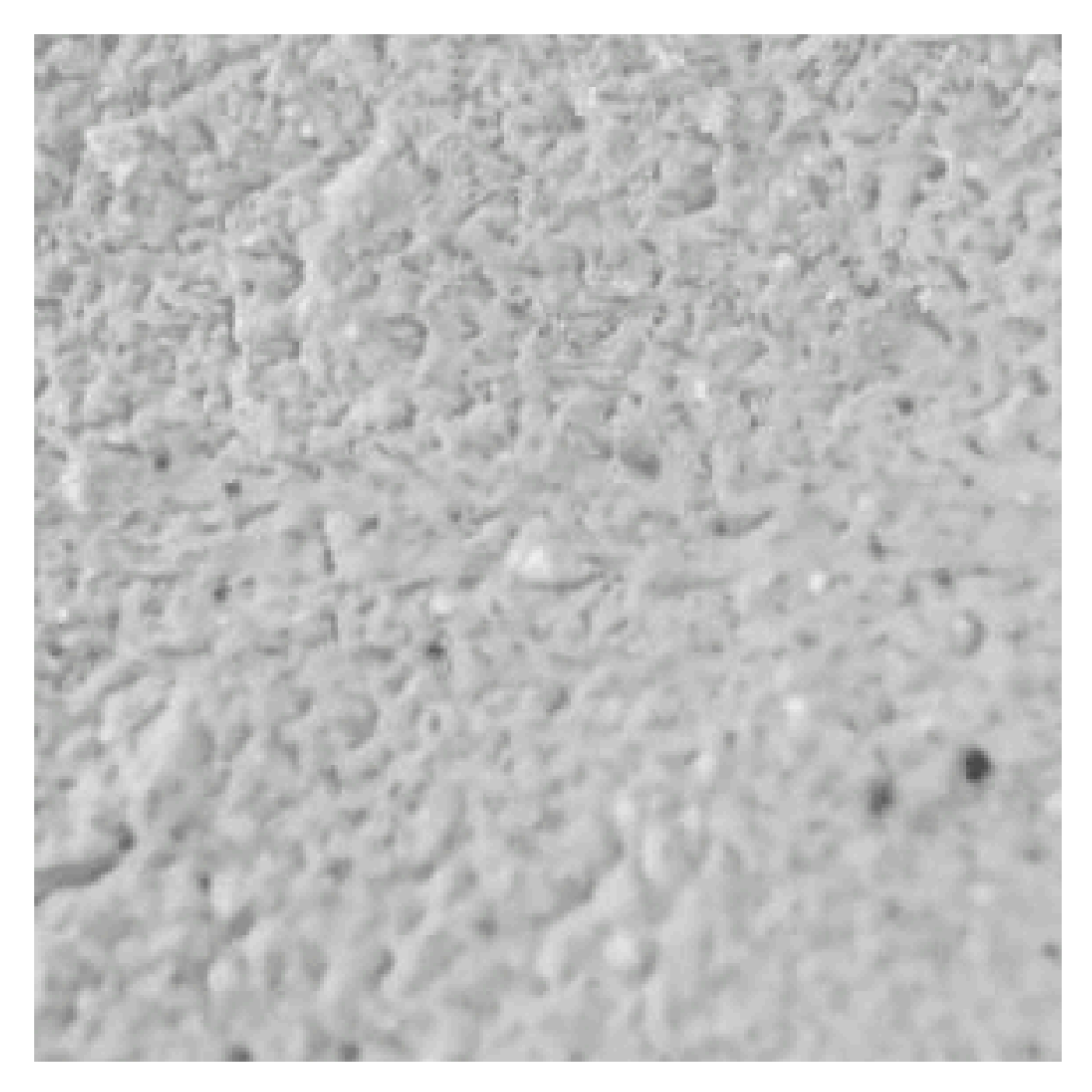
Intensity	145.38	143.62	186.13	187.77

**Table 9:** Results of orthogonal experiment.

Experiment No.	Pigment Type	Dosage (g)	Water Temperature (°C)	Setting Time (s)
1	Pigment Red 101	2 g	5 °C	390 s
2	Pigment Red 101	6 g	25 °C	240 s
3	Pigment Red 101	10 g	55 °C	120 s
4	Pigment Yellow 42	2 g	25 °C	240 s
5	Pigment Yellow 42	6 g	55 °C	120 s
6	Pigment Yellow 42	10 g	5 °C	375 s
7	Pigment Blue 27	2 g	55 °C	135 s
8	Pigment Blue 27	6 g	5 °C	375 s
9	Pigment Blue 27	10 g	25 °C	225 s

**Table 10:** Range analysis results of setting time.

Factor	Average Setting Time at Each Level (s)	Range (s)
Pigment type	Red: 250 s/Yellow: 245 s/Blue: 245 s	5 s
Pigment dosage	2 g: 255 s/6 g: 245 s/10 g: 240 s	15 s
Water temperature	5 °C: 380 s/25 °C: 235 s/55 °C: 125 s	255 s

**Table 11:** Dimensions and compressive strength of three colored specimens.

No.	Extruded Specimen	Average Width (mm)	Average Extrusion Height (mm)	Average Height After Curing (mm)	Average Compressive Strength (N)
1	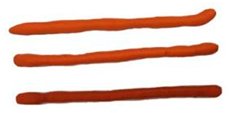	6.54 mm	5.88 mm	5.82 mm	20.03 N
2	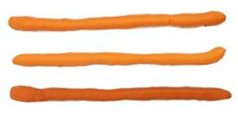	6.38 mm	5.86 mm	5.83 mm	19.93 N
3	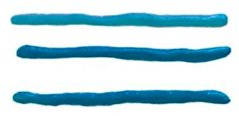	6.43 mm	5.83 mm	5.81 mm	19.13 N

**Table 12:** Color difference ΔE at each measurement point on specimens of different colors after 28 days of natural air curing.

Color/Specimen No.	ΔE_1_	ΔE_2_	ΔE_3_	ΔE_4_	ΔE_5_	ΔE_6_
Red 1	1.88	2.41	1.49	1.77	1.22	1.53
Red 2	2.10	2.59	1.68	2.01	1.41	1.70
Red 3	1.76	2.28	1.43	1.68	1.11	1.43
Yellow 1	2.18	2.84	1.77	2.12	1.28	1.61
Yellow 2	1.97	2.57	1.62	1.86	1.22	1.52
Yellow 3	2.30	2.91	1.88	2.23	1.38	1.73
Blue 1	2.00	2.52	1.56	1.88	1.20	1.50
Blue 2	1.80	2.32	1.44	1.70	1.04	1.31
Blue 3	2.12	2.63	1.72	2.01	1.26	1.57

**Table 13:** 28-day peak load test results of the specimens.

Color	Specimen 1 (N)	Specimen 2 (N)	Specimen 3 (N)	Average ± SD (N)
Red	24.54	25.13	24.06	24.58 ± 0.54
Yellow	23.92	24.48	23.46	23.95 ± 0.51
Blue	23.26	23.81	22.83	23.30 ± 0.49

**Table 14:** Comparison of raw material costs among the material developed in this study, conventional cement-based materials, and commercial colored cement.

Comparison Item	Material in This Study	Conventional Cement-Based Materials	Commercial Colored Cement
Main raw materials	Blast furnace slag, desulfurized gypsum, iron oxide pigments	Ordinary cement	Colored cement
Raw material cost (RMB/ton)	Blast furnace slag: 38–100 [45] Desulfurized gypsum: ~39 [46] Iron oxide pigment: ~6500 [47]	Cement: ~445 [48]	850–1600 [49]

## Data Availability

The original contributions presented in the study are included in the article, further inquiries can be directed to the corresponding author.
